# Transcriptome Analysis Reveals Altered Inflammatory Pathway in an Inducible Glial Cell Model of Myotonic Dystrophy Type 1

**DOI:** 10.3390/biom11020159

**Published:** 2021-01-26

**Authors:** Cuauhtli N. Azotla-Vilchis, Daniel Sanchez-Celis, Luis E. Agonizantes-Juárez, Rocío Suárez-Sánchez, J. Manuel Hernández-Hernández, Jorge Peña, Karla Vázquez-Santillán, Norberto Leyva-García, Arturo Ortega, Vilma Maldonado, Claudia Rangel, Jonathan J. Magaña, Bulmaro Cisneros, Oscar Hernández-Hernández

**Affiliations:** 1 Laboratory of Genomic Medicine, Department of Genetics, Instituto Nacional de Rehabilitación, Luis Guillermo Ibarra Ibarra, Mexico City 14389, Mexico; cuauhtli_azotla@yahoo.com.mx (C.N.A.-V.); ny3687@hotmail.com (D.S.-C.); gen.bioq@gmail.com (L.E.A.-J.); srossmary@gmail.com (R.S.-S.); nleyga06@gmail.com (N.L.-G.); maganasm@hotmail.com (J.J.M.); 2 Department of Genetics and Molecular Biology, Centro de Investigación y de Estudios Avanzados, CINVESTAV-IPN, Mexico City 07360, Mexico; jose.hernandezh@cinvestav.mx (J.M.H.-H.); bcisnero@cinvestav.mx (B.C.); 3 Escuela Nacional de Ciencias Biologicas-Instituto Politécnico Nacional, Mexico City 11340, Mexico; 4 Computational and Integrative Genomics Laboratory, Instituto Nacional de Medicina Genómica, Mexico City 14610, Mexico; jorge.pena@cgu.edu (J.P.); crangel@inmegen.gob.mx (C.R.); 5 Institute of Mathematical Sciences, Claremont Graduate University, Claremont, CA 91711, USA; 6 Epigenetics Laboratory, Instituto Nacional de Medicina Genomica, Mexico City 14610, Mexico; kivs09@gmail.com (K.V.-S.); vilmaml@gmail.com (V.M.); 7 Department of Toxicology, Centro de Investigación y de Estudios Avanzados, CINVESTAV-IPN, Mexico City 07360, Mexico; arortega@cinvestav.mx; 8 School of Engineering and Sciences, Department of Bioengineering, Tecnológico de Monterrey-Campus, Mexico City 14380, Mexico

**Keywords:** myotonic dystrophy type 1, inducible cell models, RNA foci, gene expression, microarrays

## Abstract

Myotonic dystrophy type 1 (DM1), the most frequent inherited muscular dystrophy in adults, is caused by the CTG repeat expansion in the 3′UTR of the *DMPK* gene. Mutant *DMPK* RNA accumulates in nuclear foci altering diverse cellular functions including alternative splicing regulation. DM1 is a multisystemic condition, with debilitating central nervous system alterations. Although a defective neuroglia communication has been described as a contributor of the brain pathology in DM1, the specific cellular and molecular events potentially affected in glia cells have not been totally recognized. Thus, to study the effects of DM1 mutation on glial physiology, in this work, we have established an inducible DM1 model derived from the MIO-M1 cell line expressing 648 CUG repeats. This new model recreated the molecular hallmarks of DM1 elicited by a toxic RNA gain-of-function mechanism: accumulation of RNA foci colocalized with MBNL proteins and dysregulation of alternative splicing. By applying a microarray whole-transcriptome approach, we identified several gene changes associated with DM1 mutation in MIO-M1 cells, including the immune mediators *CXCL10*, *CCL5*, *CXCL8*, *TNFAIP3,* and *TNFRSF9*, as well as the microRNAs miR-222, miR-448, among others, as potential regulators. A gene ontology enrichment analyses revealed that inflammation and immune response emerged as major cellular deregulated processes in the MIO-M1 DM1 cells. Our findings indicate the involvement of an altered immune response in glia cells, opening new windows for the study of glia as potential contributor of the CNS symptoms in DM1.

## 1. Introduction

Myotonic Dystrophy type 1 (DM1), an inherited neuromuscular disorder, is the most common form of muscular dystrophy in adults, with a prevalence between 0.5–18.1 per 100,000 [1]. DM1 is caused by a CTG repeat expansion located in the 3′ untranslated region (UTR) of the myotonic dystrophy protein kinase (*DMPK*) gene. The CTG tract consists of 5 to 37 repeats in healthy individuals, but it is expanded to more than 50 CTG repeats in DM1 patients [2,3,4]. DM1 is a multisystemic disease with a broad clinical presentation including myotonia, muscle weakness, heart conduction abnormalities, insulin resistance, endocrine defects, frontal balding, cataracts, and central nervous system (CNS) alterations [5]. The neuronal features exhibited by patients with DM1 depend on the age of onset and size of the CTG tract [6,7]. Specifically, intellectual disability is present in the congenital form of DM1; while learning difficulties, speech and language delay, reduced IQ-values accompanied with attention deficit hyperactive disorder, and autism spectrum are frequent in infantile- and juvenile-onset DM1 [8,9]. Apathy and reduced initiative, attention deficit, excessive daytime sleepiness, and fatigue are commonly present in the adult form [7]. Finally, important neuropsychological signs and symptoms, such as anxiety, hostility, depression, low esteem and paranoia, are also frequent in DM1 patients [10,11]. Overall CNS symptomatology has a negative impact on the quality of life of patients [12].

DM1 pathogenesis is mediated by an RNA gain-of-function mechanism, through accumulation of mutant *DMPK* mRNA aggregates in the nucleus of affected cells [13,14,15]. This in turn alters directly or indirectly numerous cell functions, including transcription regulation, RNA polyadenylation, translation, microRNA (miRNA) processing, and alternative splicing [16,17,18,19,20]. In addition, the detection of small *DMPK* RNAs expressed in the antisense direction [21], as well as the occurrence of repeat-associated non-ATG translation [22,23], which results in the accumulation of homopolymeric proteins, have added further complexity to molecular pathogenesis of DM1. Perturbation of alternative splicing through sequestration of muscleblind-like (MBNL) family of factors by mutant *DMPK* RNA and upregulation of CUG/Elav-like (CELF) proteins [24,25] are the best described pathogenic mechanisms in DM1. In fact, numerous mis-splicing events have been described in genes expressed in DM1 brain tissues implicated in its CNS symptomatology, including *GRIN1/NMDAR1* exons 5 and 21, *MAPT* exons 2–3, 10, *APP* exon 8, *MBNL1/2* exon 7, *CAMK2D* exons 14–15, and *SORBS* exon 26 [14,26,27].

In spite of the role of glial cells in essential brain functions, including synapsis, inflammation response, and neurotransmission [28], their implication in DM1 neuropathology just began to be addressed. Recent evidence shows that cortical astrocytes contain greater abundancy of ribonuclear foci than neurons in the brain of DM1 transgenic mice [26]. In addition, *DMPK* transcript levels are higher in astrocytes and glia-derived cell lines, compared to neurons [29]; and finally, glutamate excitotoxicity associated with downregulation of glutamate transporter 1 (GLT1) is observed in Bergmann glia of DM1 mice [30]. All these observations suggest that defective neuron-glia interactions, which are fundamental for proper brain function, might be an important contributor to DM1 brain pathophysiology. 

To gain insight into the cellular and molecular pathways primarily affected by the DM1 mutation in glial cells, in this study, we generated a DM1 model derived from human retinal Müller glial cells (MIO-M1) [31], which express a mutant *DMPK* RNA carrying 648 CUG repeats in an inducible manner [MIO-M1 CTG_(648)_]. Validation experiments indicated the occurrence of an RNA toxicity mechanism in the established model, characterized by altered alternative splicing and nuclear co-localization of RNA foci with MBNL1/2 splicing factors. By conducting transcriptome analysis of MIO-M1 CTG_(648)_ cells, we observed altered expression levels of numerous genes upon expression of the CTG repeat expansion, including immune mediators and microRNA genes. A gene ontology analysis approach led us to the identification of inflammatory pathway and immune response as the major cellular deregulated processes in MIO-M1 CTG_(648)_ cells. Considering the role of glial cells as modulators of neuroinflammation, these results open new avenues for the study of DM1 brain pathophysiology associated with defective glia.

## 2. Materials and Methods 

### 2.1. Cell Culture

MIO-M1 cells (Moorfields/Institute of Ophthalmology, University College London) were grown in presence of Dulbecco’s Modified Eagle Medium (DMEM) (Invitrogen, Carlsbad, CA, USA), supplemented with 10% fetal bovine serum (FBS), 100 U/mL penicillin and 100 μg/mL streptomycin. Cultures were maintained at 37 °C in a 5% humidified CO_2_ atmosphere. 

### 2.2. Generation of MIO-M1 DM1 Cell Model

MIO-M1 cell line was stably transfected with the pCMV-Tet3G plasmid (Clontech, Mountain View, CA, USA). After 3 weeks on geneticin (G418) selection, independent clones were assayed with the Dual-Luciferase Reporter Assay System (Promega Corporation, Madison, WI, USA). The clone with the high doxycycline induction and the low basal luciferase activity (MIO-M1-CMV-Tet) was selected. Simultaneously, fragments of DMPK minigene containing exons 11–15, carrying an expanded CTG tract, or 0 CTG repeat tract as control, were excised by NheI digestion from DT960 and DMPKS plasmids [32], respectively. Expanded or control DMPK minigene fragments were ligated to the equally restricted pTRE3G plasmid (Clontech, Mountain View, CA, USA) to generate the CTG expanded and control Tet responsive constructs. Then, MIO-M1-CMV-Tet cells were stably transfected with the responsive plamids and selected in the presence of 350 μg/mL G418 and 0.35 μg/mL puromycin to obtain the control MIO-M1-CTG_(0)_ and the mutant MIO-M1-CTG_(648)_ cells. The control MIO-M1-CTG_(0)_ and the mutant MIO-M1-CTG_(648)_ established cell lines were subsequently cultured in presence of 10% FBS tetracycline-free.

### 2.3. Genotyping

Genomic DNA was extracted from cell cultures and genotyping was performed by triplet repeat-primed polymerase chain reaction (TP-PCR) and capillary electrophoresis on a 3730 xl DNA analyzer (Applied Biosystems, Foster City, CA, USA) as previously described [33]. Estimation of expanded allele size was carried out by Small Pool PCR (SP-PCR) as described by Tomé S. et al. [34]. 

### 2.4. RNA Isolation, Retrotranscription and Polymerase Chain Reaction (RT-PCR) 

Total RNA was isolated from cell cultures using TRIzol Reagent (Invitrogen, Carlsbad, CA, USA). RNA integrity was analyzed by gel electrophoresis. A NanoDrop 2000 spectrophotometer (NanoDrop Technologies, Wilmington, DE, USA) was used for quantification and purity determination. Total RNA (1 µg) was used to prepare cDNA by using the High-Capacity cDNA reverse transcription kit according to manufacturer’s protocol (Thermo Fisher Scientific. Waltham, MA, USA). PCR reactions were carried out in 12.5 μL total volume by using the Platinum Taq DNA pol (Invitrogen, Carlsbad, CA, USA). The percentage of exon inclusion was calculated as [exon inclusion band/(exon inclusion band + exon exclusion band)] x 100 [26]. Oligonucleotide primers used for RT-PCR analysis to detect DMPK/GAPDH expression and MBNL1/2 alternative splicing patterns are indicated in Table S1.

### 2.5. RNA Fluorescence In Situ Hybridization (RNA-FISH) and Immunofluorescence

Cells on coverslips were fixed with 4% paraformaldehyde for 10 min at room temperature, then permeabilized with cold 2% acetone for 5 min, and overnight incubation in 70% ethanol. Cells were prehybridized in 30% formamide, 2X SSC buffer for 10 min at room temperature and then incubated for 2 h in a humidified chamber at 37 °C with hybridization buffer [2X SSC, 40% formamide, 0.02% BSA, 2mM vanadyl ribonucleoside (Sigma-Aldrich, St. Louis, MO, USA), 66 µg/mL yeast tRNA (Sigma-Aldrich, St. Louis, MO, USA) and 1 ng/µl Cy3-conjugated CAG_(6)_ probe]. Preparations were washed in prehybridization buffer at 45 °C for 30 min, twice in 1X SSC at room temperature, and once in PBS. Cells were mounted on microscope slides with Vectashield antifade medium containing diamino-2-phenylindole (DAPI) (Vector Labs., Burlingame, CA, USA). For combine immunofluorescence and FISH, cells were incubated in 3% BSA for 15 min at room temperature after the post-hybridization wash step of the RNA FISH procedure, then incubated overnight at 4 °C with the primary anti-MBNL1 or MBNL2 antibodies (Abcam, Cambridge, UK. ab45899 and ab171551, respectively). After washing three times with PBS, preparations were incubated 1h at room temperature with fluorescein-conjugated anti-rabbit antibody (Vector Labs., Burlingame, CA, USA), counterstained with DAPI and mounted with Vectashield.

### 2.6. Microarray Processing

MIO-M1-CTG_(0)_ and MIO-M1-CTG_(648)_ cells were cultured in absence (-Dox) or presence (+Dox) of doxycycline (1 μg/mL) during 8 days, replacing culture medium every 48 h. For each of the four experimental groups, 3 biological replicates were used. Total RNA was extracted using RNeasy Mini kit (Qiagen, Valencia, CA, USA) and RNase-Free DNase Set, according to the manufacturer’s protocol. The RNA concentration was determined using a NanoDrop 2000 spectrophotometer (NanoDrop Technologies, Wilmington, DE, USA), and the RNA integrity number (RIN) was determined in an Agilent 2100 Bioanalyzer (Agilent Technologies Inc, Santa Clara, CA, USA). Only RNA samples with a RIN > 9 were employed. Sample preparation was carried out as described in the Affymetrix GeneChip WT Plus Reagent Kit User Manual (Affymetrix Inc., Santa Clara, CA, USA). Briefly, 100 ng of total RNA were used for synthesize first-strand cDNA followed by a reaction that uses DNA polymerase I and RNase H to simultaneously degrade the RNA and synthesize second-strand cDNA, which was subsequently converted to cRNA by in vitro transcription. Finally, the sense-strand cDNA was obtained by reverse transcription of the cRNA, loaded onto Clariom D Arrays for human samples (Thermo Fisher Scientific. Waltham, MA, USA) and incubated for 16 h at 45 °C on the GeneChip Hybridation Oven 645 (Affymetrix Inc., Santa Clara, CA, USA). After hybridization, arrays were washed and stained using the GeneChip Fluidics Station 450 followed by scanning with the GeneChip Scanner 3000 7G (Affymetrix Inc., Santa Clara, CA, USA). Microarray experiment data have been deposited in NCBI’s Gene Expression Omnibus database (GSE164057).

### 2.7. Microarrays Data Analysis

The raw intensity values were background corrected and normalized using Robust Multiarray Average (RMA) method [35] and Quantile Normalization [36], respectively. Statistical linear model with arbitrary coefficients was used to determine differential expression, contrasts of interest were analyzed using the Bioconductor library limma [37,38]. Corrections for multiple comparisons were applied using false discovery rate (FDR). Differentially expressed genes were considered as log fold change (FC) >0.5 and *p* val < 0.005.

### 2.8. Functional Genomic Analysis

Gene ontology enrichment analyses were performed by Gene set enrichment analysis (GSEA) [39], DAVID (http://david.abcc.ncifcrf.gov), Key Pathway Advisor (KPA version 17.4) and MetaCore (version 19.2). Overlapping list of differentially expressed genes, founded in contrasts A and B, was used to perform DAVID, KPA and MetaCore analyses. GSEA was performed to measure the enrichment of annotated gene sets which represent biological altered processes in MIO-M1-CTG_(648)_ Dox-induced cells compared with non-induced MIO-M1-CTG_(648)_ cells. Analysis was run using default settings and 1000 permutation with a weighted enrichment statistic and a signal-to-noise metric for gene ranking. Gene sets were downloaded from the MSigDB database. Normalized enrichment scores (NES) and *p*-values were calculated to measure differences in gene set size as well as correlation with other gene signatures. KPA and MetaCore were used to identify canonical pathways having statistically significant changes. Top pathways and functions were scored and sorted by predicted activation and by the number of molecules involved. MicroRNA target prediction was performed by using mirDIP 4.1 integrative [40] and miRDB [41] online databases. DAVID and ShinyGO [42] tools were used to executed enrichment analysis of predicted miRNA targets.

### 2.9. TaqMan Validation Assays

The expression of selected transcripts was evaluated by retrotranscription and quantitative PCR (RT-qPCR) by using TaqMan probe-based assays (Applied Biosystems, Foster City, CA, USA). Total RNA extraction and retrotranscription were performed as described in the Microarray processing section. PCR was performed in a 20 μL total volume reaction by mixing 100 ng cDNA, 2X TaqMan Universal Master Mix II with UNG (Applied Biosystems, Foster City, CA, USA), 1μL of specific target TaqMan assay probe, and 1 μL of primer limited probe for the endogenous control glyceraldehyde-3-phosphate deshydrogenase (GAPDH). Amplification was conducted at 50 °C for 2 min, 95 °C for 10 min as initial step followed by 95 °C for 15 sec, 60 °C for 1min for 40 cycles on a StepOne Real Time PCR System (Applied Byosistems, Foster City, CA, USA). Relative gene expression was calculated using the 2^ΔΔCT^ method. 

### 2.10. Statistical Analysis

When two groups were compared, an unpaired Student’s t-test for statistical significance were performed. The significance level was set at *p* < 0.05. Data were expressed as mean ± standard error of the mean (SEM). The statistical software GraphPad Prism package was used for calculations.

## 3. Results

### 3.1. Generation and Characterization of a MIO-M1 Glial Cell-based Model for DM1

**Figure 1:**
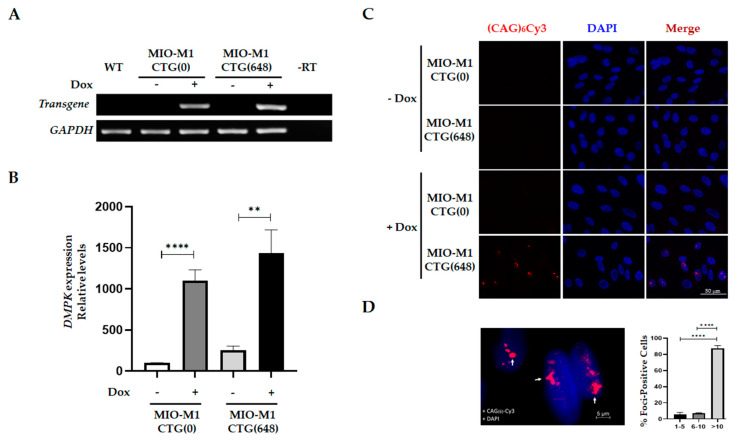
Inducible expression of mutant *DMPK* minigene elicits nuclear foci in MIO-M1-CTG_(648)_ cells. (**A**) Expression of exogenous *DMPK* minigene was analyzed by RT-PCR in MIO-M1-CTG_(0)_ and MIO-M1-CTG_(648)_ cells, before (-) and after (+) Dox (1 µg/mL) induction, using *GAPDH* as endogenous control. WT: RT-PCR using cDNA from MIO-M1 wild type cells, -RT: RT-PCR using cDNA prepared in absence of retro-transcriptase enzyme. (**B**) Quantification of *DMPK* expression indicates comparable levels of transgene between MIO-M1-CTG_(0)_ and MIO-M1-CTG_(648)_ cells after (+) Dox induction. Values are the mean ± SEM of three independent experiments, with significant differences determined by unpaired t-test, **** denotes *p* < 0.0001, ** denotes *p* < 0.005. (**C**) In situ hybridization of CUG repeats using a (CAG)_6_ Cy3 probe were carried out in MIO-M1-CTG_(0)_ and MIO-M1-CTG_(648)_ cells in absence (-Dox) and presence (+Dox) of doxycycline. Cells were counterstained with DAPI for nuclei visualization prior to being analyzed by confocal microscopy. Representative single typical optical Z-sections were selected to show the presence nuclear foci. (**D**) 15X magnification of representative MIO-M1-CTG_(648)_ cells cultured under Dox induction (left), and quantitation of foci-positive cells (right). Data in the graph are the mean ± SEM of triplicate experiments (*n* = 200 cells), **** denotes *p* < 0.0001.

To analyze the effect of DM1 mutation on glial cell physiology, we generated MIO-M1-derivative cell lines expressing a *DMPK* minigene harboring 0 CTG repeats [MIO-M1-CTG_(0)_] or an expanded repeat [MIO-M1-CTG_(648)_], in an inducible manner (Figure S1A). Stable incorporation of the *DMPK* minigene into cells was confirmed by genotyping using TP-PCR and SP-PCR assays. A ladder pattern of amplified fragments was obtained from MIO-M1-CTG_(648)_ cells after TP-PCR analysis, unlike MIO-M1-CTG_(0)_ cells, which lacked this characteristic pattern (Figure S1B). Confirming this result, SP-PCR experiments on MIO-M1-CTG_(648)_ cells resulted in the amplification of a DNA fragment that corresponds to 648 CTG repeats tract, while no amplified expanded fragment was obtained from MIO-M1-CTG_(0)_ cells (Figure S1C). Together, these results demonstrated that control and mutant *DMPK* minigene was successfully inserted into the genome of MIO-M1 CTG_(0)_ and MIO-M1 CTG_(648)_ cells, respectively. To analyze *DMPK* minigene expression in MIO-M1-CTG_(0)_ and MIO-M1-CTG_(648)_ cells, RT-PCR experiments were performed, using a forward primer that exclusively amplifies *DMPK* minigene transcripts. Both MIO-M1-CTG_(0)_ and MIO-M1-CTG_(648)_ cells expressed the exogenous *DMPK* RNA after exposure to doxycycline (+Dox) for 3 days, with no detectable basal expression in the absence of Dox (-Dox) (Figure 1A), demonstrating the specificity of the inducible system. In addition, *DMPK* expression levels were comparable between MIO-M1-CTG_(0)_ and MIO-M1-CTG_(648)_ cells, as shown by RT-qPCR analysis (Figure 1B). Since it has been extensively described that mutant *DMPK* RNA induces ribonucleoprotein foci in DM1 tissues/cells, we asked whether Dox-induced MIO-M1-CTG_(648)_ cells contained nuclear foci of mutant *DMPK* RNA. RNA-FISH experiments showed that mutant *DMPK* RNA foci were present in MIO-M1-CTG_(648)_ cells upon 3 days of Dox induction, unlike MIO-M1-CTG_(0)_ cells, where no nuclear foci was detected (Figure 1C). Quantitative analysis revealed that 75% of cells had nuclear foci, out of these, 87.5% containing >10 foci, 7.0% between 6–10 foci and 5.5% between 1–5 foci (Figure 1D). 

**Figure 2:**
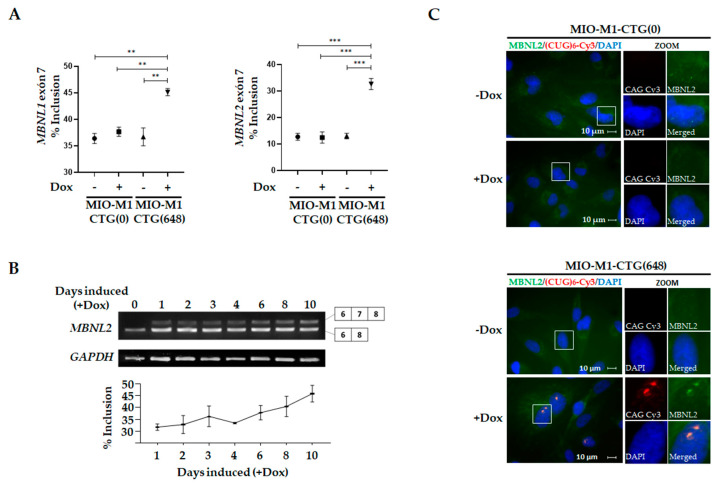
Alternative splicing of exon 7 in *MBNL1* and *MBNL2* mRNAs is dysregulated in MIO-M1-CTG_(648)_ cells. (**A**) The inclusion rate of *MBNL1* and *MBNL2* exon 7 was assessed by RT-PCR in MIO-M1-CTG_(0)_ cells and MIO-M1-CTG_(648)_ cells, before (-) and after (+) Dox (1 µg/mL) induction for 3 days. Data shown are means ± SEM of three independent experiments, with *p* values indicating significance differences (unpaired t-test), ** represents *p* < 0.005, *** denotes *p* < 0.0005. (**B**) *MBNL2* exon 7 inclusion increased progressively after Dox induction for the indicated number of days in MIO-M1-CTG_(648)_ cells. *GAPDH* expression was used as endogenous control (**C**) RNA FISH [CAG)6- Cy3] and immunofluorescence (MBNL2) showed colocalization of mutant RNA with MBNL2 exclusively in MIO-M1 CTG_(648)_ upon Dox induction (+Dox). Cells were counterstained with DAPI for nuclei visualization prior to being analyzed by confocal microscopy. 4.5X magnification (ZOOM) is showed for each condition. Representative single typical optical Z-sections were selected to show the presence nuclear foci.

Dysregulation of alternative splicing is a central pathogenic mechanism broadly described in DM1. In order to explore this process in our model, we analyzed the exon 7 inclusion rate of *MBNL1* and *MBNL2*, two DM1-associated mis-splicing events [43,44,45]. As anticipated, higher percentage of splicing inclusion for *MBNL1* exon 7 was observed in Dox-induced MIO-M1-CTG_(648)_ cells (45.1%) compared to non-induced MIO-M1-CTG_(648)_ (36.7%) and to both non-induced and Dox-induced MIO-M1-CTG_(0)_ cells (36.4% and 37.7 %, respectively) (Figure 2A left panel and Figure S2A). Similarly, MIO-M1-CTG_(648)_ cells showed higher *MBNL2* exon 7 inclusion (32.7%) compared to non-induced MIO-M1-CTG_(648)_ cells (13.0%), and to both non-induced and Dox-induced MIO-M1-CTG_(0)_ cells (12.8% and 12.5%, respectively), (Figure 2A right panel and Figure S2A). Notably, the sustained Dox treatment further increased the aberrant splicing of *MBNL2* exon 7 over time in the MIO-M1-CTG_(648)_ cells (Figure 2B), likely due to the nuclear foci accumulation. As functional depletion of MBNLs, by its sequestration into the nuclear foci, causes numerous mis-splicing events, we were prompted to examine whether MBNL1/MBNL2 proteins are co-localized with RNA foci in MIO-M1-CTG_(648)_ cells. RNA-FISH coupled with immunofluorescence assays revealed that both, MBNL1 (Figure S2B) and MBNL2 (Figure 2C) co-localize with CUG RNA foci in MIO-M1-CTG_(648)_ cells upon Dox induction, while MIO-M1-CTG_(0)_ cells displayed diffuse MBNL protein staining, supporting the idea that mutant RNA is altering the MBNLs function. Collectively, these results demonstrated the occurrence of the DM1 molecular hallmarks in the established cell model, indicative of the existence of an RNA toxicity mechanism in MIO-M1-CTG_(648)_ cells.

### 3.2. Effects of DM1 Mutation on Global Gene Expression in MIO-M1-CTG_(648)_ Cells

**Figure 3:**
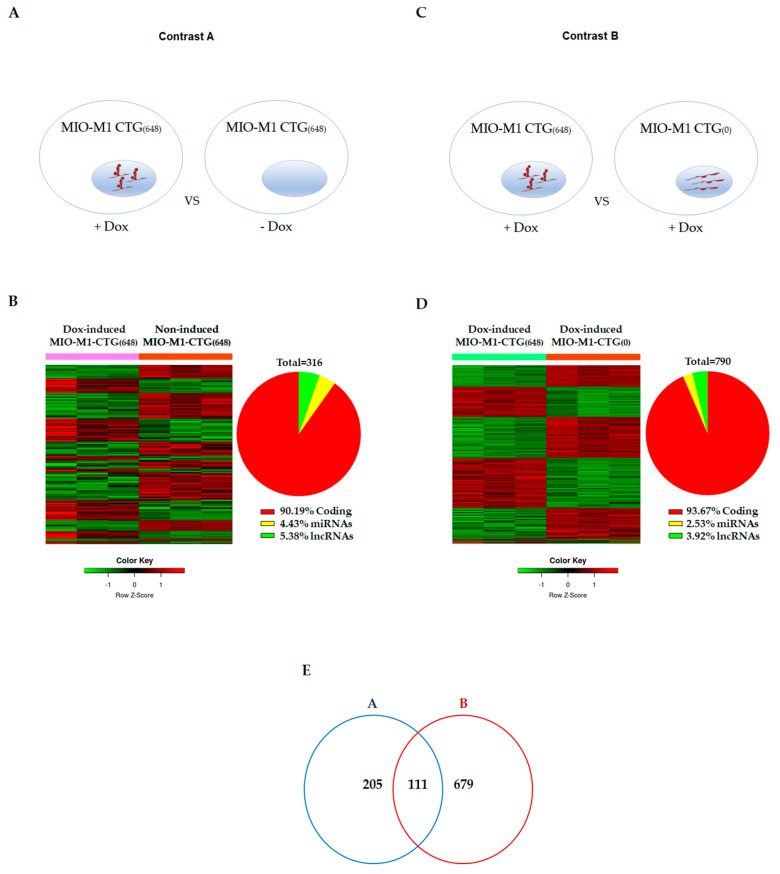
Transcriptome changes in MIO-M1-CTG_(648)_ cells expressing DM1 mutation. (**A**) Contrast A gene set was obtained by the comparison of profiles between MIO-M1-CTG_(648)_ Dox-treated (+Dox) cells vs MIO-M1-CTG_(648)_ non-induced (-Dox) cells. Gene changes caused by Dox treatment were first identified contrasting MIO-M1-CTG_(0)_ Dox-induced cells vs MIO-M1-CTG_(0)_ non-induced cell profiles, and then were discharged from this data set (**B**) Heat map representation (left) of the 187 up-regulated (red) and 129 down-regulated (green) genes from a total of 316 differentially expressed genes between MIO-M1 CTG_(648)_ cells with and without Dox-induction (contrast A). Pie chart (right) illustrates the percentage of coding and non-coding genes determined by this comparison. (**C**) Contrast B gene set was obtained by the comparison of profiles between MIO-M1-CTG_(648)_ Dox-treated cells vs MIO-M1-CTG_(0)_ Dox-induced cells (+Dox). Gene changes derived from gene insertion site were first identified contrasting gene profiles of MIO-M1-CTG_(648)_ vs MIO-M1-CTG_(0)_ both non-induced (-Dox) cells, and subsequently discharged from this data set. (**D**) Heat map (left) illustrating the 449 up-regulated (red) and 342 down-regulated (green) genes from a total of 790 gene changes between Dox-induced MIO-M1-CTG_(648)_ cells and Dox-induced MIO-M1-CTG_(0)_ cells (contrast B). Pie chart (right) showing the % of coding and non-coding genes revealed by this contrast. (**E**) Venn diagram showing overlapping (111) and non-overlapping (884) gene changes between contrast A and B.

Several transcription-associated processes are altered in DM1. Thus, it is reasonable to expect global transcriptional changes in our DM1 model. To evaluate this, we determined the gene expression differences between Dox-induced MIO-M1-CTG_(648)_ and non-induced MIO-M1-CTG_(648)_ cells (Figure 3A, Contrast A). We first compared the gene expression profile between MIO-M1-CTG_(0)_ Dox-treated cells and MIO-M1-CTG_(0)_ non-induced cells, in order to remove those genes dysregulated by the Dox treatment. Contrast A led us to identify 316 differentially expressed genes, with 187 up-regulated (59.17%) and 129 down-regulated (40.82%) genes. From this, 285 corresponded to coding (90.19%) and 31 to non-coding genes (9.81%), including 14 miRNAs (4.43%) and 17 long non-coding RNAs (5.38%) (Figure 3B). In parallel, a second contrast (Figure 3C, Contrast B) was analyzed. In this case, we first removed differential effects caused by the transgene insertion site, and then, we compared the transcriptome of Dox-induced MIO-M1-CTG_(648)_ cells versus that of Dox-induced MIO-M1-CTG_(0)_ cells. In contrast B, 790 differentially expressed genes were identified, with 449 up-regulated (56.84%) and 342 down-regulated (43.29%) genes. From this, 740 corresponded to coding (93.67%) and 51 to non-coding genes (6.46 %), including 20 miRNAs (2.53%) and 31 long non-coding RNAs (3.92%) (Figure 3D). A total of 111 differential expressed genes were overlapped in both contrasts, representing 90.9 % coding and 9.1 % non-coding gene changes (Figure 3E and Tables S2 and S3). The ten most up and down differentially expressed genes, that were common in both contrasts (A and B) are shown in Table 1.

In order to confirm the validity of the microarray data, the expression levels of *CXCL10*, *TNFRSF9* and *TNFAIP3*, identified as upregulated genes, and *PLXDC1* and *TSPAN18*, identified as downregulated genes, were validated by TaqMan probe-based assays on three independent culture replicates. Levels of *CORO2B*, a gene with any observed changes, were also validated (Figure S3). The expression alteration and directionality of all the transcripts analyzed was confirmed (Table 2 and Figure S3). Collectively, these results showed that DM1 mutation is altering gene expression regulation in MIO-M1-CTG_(648)_ cells.

### 3.3. Gene Ontology Analysis Reveals Altered Inflammatory Processes in MIO-M1-CTG_(648)_ Cells

**Figure 4:**
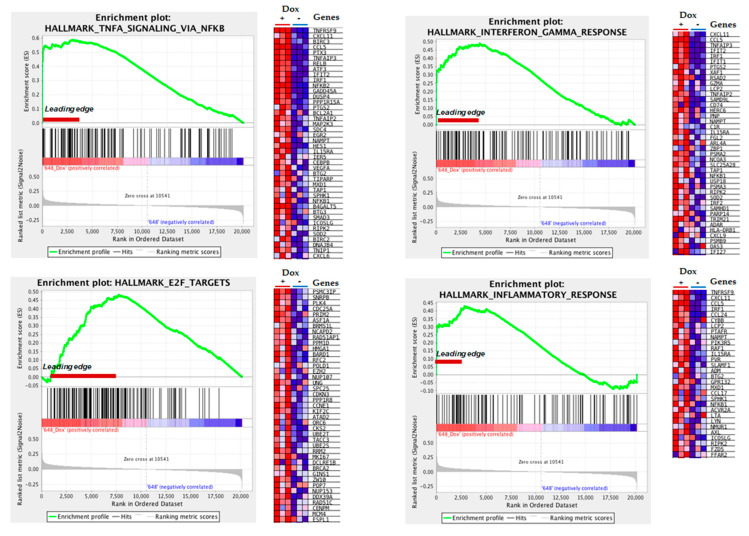
GSEA revealed inflammation altered processes associated with DM1 mutation in MIO-M1 cells. Enrichment plots of the four highly significant hallmark gene-sets are showed. Leading edge subset denote the more biologically important genes, which are showed in the corresponding heat map for Dox-induced MIO-M1 CTG_(648)_ cells (+Dox) and non-induced MIO-M1 CTG_(648)_ cells (−Dox) as control. Red: up-regulated, blue: down-regulated. Normalized enrichment score (NES) and false discovery rate (FDR) are indicated.

To understand the functional alterations behind the gene changes in MIO-M1-CTG_(648)_ cells, we performed gene ontology (GO) enrichment analyses using different bioinformatic tools. By GSEA approach, we identified the TNF-α via NFκB signaling pathway, the INF-γ response mechanism, the E2F targets, and the inflammatory response as the most gene set enriched hallmarks in MIO-M1-CTG_(648)_ Dox-induced cells (Figure 4). Likewise, we identified the chemokine mediated signaling pathway as the most significantly overrepresented biological process (Table 3, GSEA Resource/BP category). For cellular component category, the spliceosomal complex was overrepresented (Table 3, GSEA Resource/CC category). Similarly, the most enriched molecular function was the chemokine receptor binding (Table 3, GSEA Resource/MF category). The list of the most enriched gene set and processes revealed by GSEA, DAVID, and KPA tools is showed in Table 3. Remarkably, GO enrichment analysis highlighted immune response-related processes in MIO-M1-CTG(648) Dox-induced cells. Additional processes were also recognized, included the non-coding RNA (ncRNA) transcription, progression through the cell division cycle (G2/M Checkpoint), RNA splicing via transesterification reactions, and the spliceosome pathway (Figure S4).

**Figure 5:**
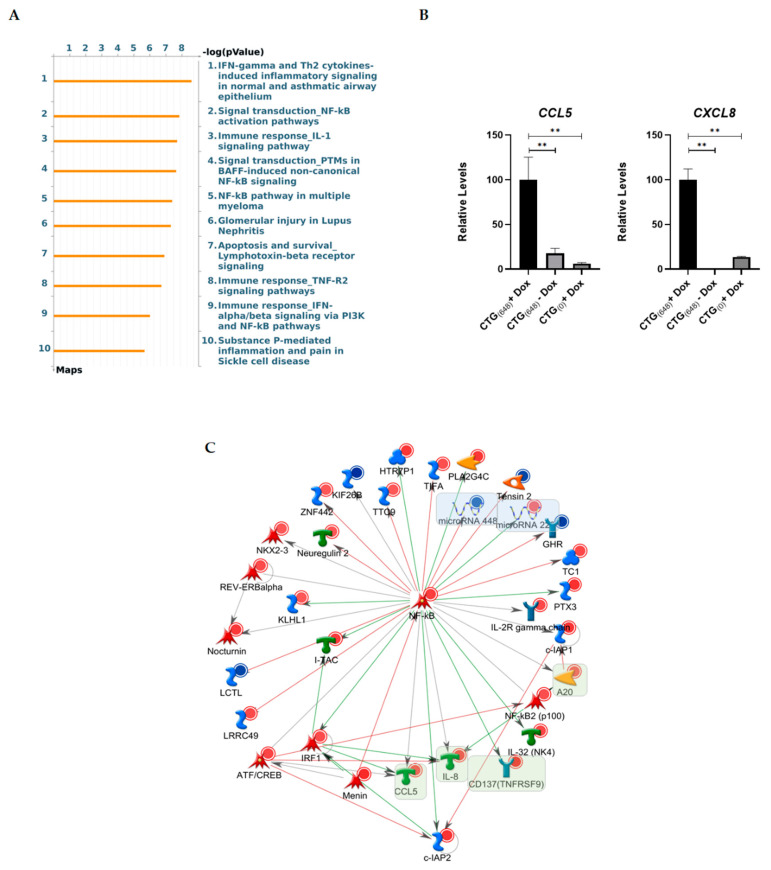
Functional enrichment analysis by MetaCore showed involvement of immune system pathways in MIO-M1-CTG_(648)_ cells (**A**). Top 10 enriched Pathway Maps sorted by statistical significance -log (*p* value) of the findings. (**B**) Up-regulation of *CCL5* and *CXCL8* (*IL-8*) were validated by TaqMan assays in Dox-induced MIO-M1-CTG_(648)_ cells. Data shown are means ± SEM from three independent experiments. Unpaired t-test was used to determine statistical significance. ** denotes *p* > 0.005 (**C**) Network analysis of dysregulated genes shows connection with NFκB transcription factor. *CCL5*, *CXCL8* (*IL-8)*, *TNFRSF9*, and *TNFAIP3* (*A20*), indicated in green boxes, are positively NFκB-regulated targets. miR-448 and miR-222, showed in blue rectangles, are negatively (red line) and positively (green line) NFκB-regulated targets, respectively.

GO enrichment results were supported by findings obtained with MetaCore pathway analysis. The signal transduction NF-κB activation pathway and the immune response IL-1 signaling pathway were identified among the top 10 Pathway Maps (Figure 5A). IL-1 is a pro-inflammatory cytokine which activates a broad range of transcription factors, including IRF1 and NFκB, thus, inducing a spectrum of immune and inflammatory responses (Figure S5). Since IL-1 pathway positively regulates the expression of the chemokines *CXCL8 (IL-8*) and *CCL5* [46,47], we asked whether MIO-M1-CTG_(648)_ cells overexpressed these two chemokine transcripts upon Dox-induction. As predicted, RT-qPCR experiments demonstrated up-regulated levels of *CCL5* and *CXCL8* in Dox-induced MIO-M1-CTG_(648)_ cells compared with non-induced MIO-M1-CTG_(648)_ and Dox-induced MIO-M1-CTG_(0)_ cells (Figure 5B), thus, confirming the microarray data (see Table 1). Subsequently, we performed a MetaCore network analysis to identify important transcription factors connected with the query genes in MIO-MI-CTG_(648)_ Dox-treated cells. This approach led us to identify numerous NFκB gene targets, including *CCL5*, *IL-8*, *TNFRSF9*, *CXCL10*, and interestingly, miR-222 and miR-448, two dysregulated miRNAs in MIO-MI-CTG_(648)_ Dox-treated cells (Figure 5C and Table 4).

**Figure 6:**
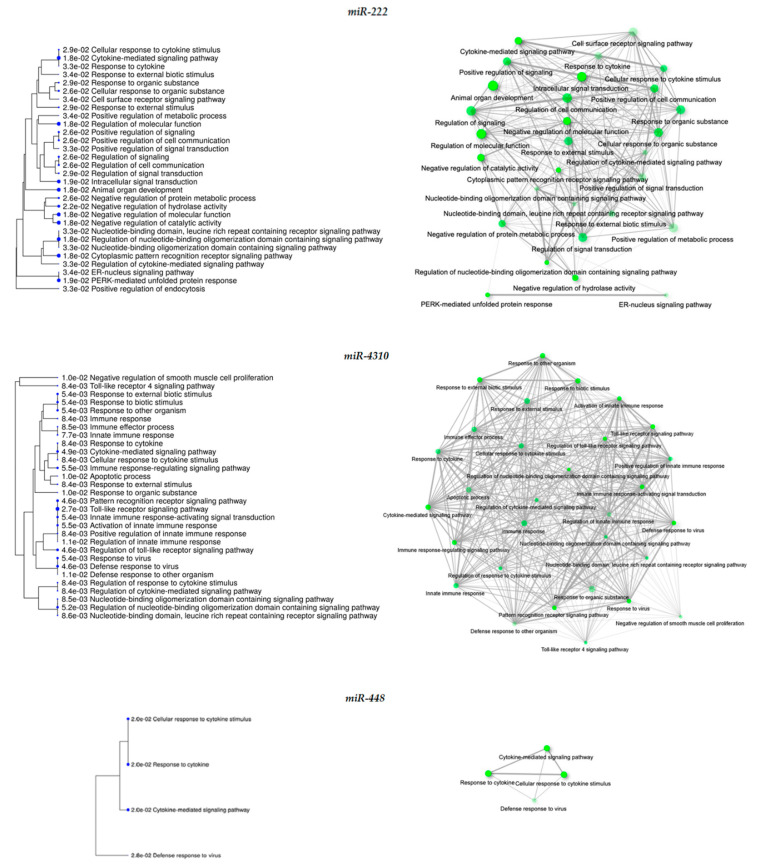
Enriched biological processes for the indicated dysregulated miRNA targets in MIO-M1-CTG_(648)_ Dox-induced cells. Hierarchical clustering trees (**left**), and network diagrams (**right**) were performed using ShinyGO server. Nodes (circles) on networks, denote enriched processes; lines denote interactions among genes that are common between processes.

In addition to miR-222 and miR-448, we detected altered expression of miR-4288, miR-103a-1, miR-298, and miR-4310 in both contrasts A and B. To get insight into the possible function of these dysregulated miRNAs could have in MIO-M1-CTG_(648)_ Dox-induced cells, we first determined their transcript targets, filtered by nervous system expression, in the miRDB data base. The list of targets was then subjected to GO enrichment analyses. Nervous system development was the most significantly enriched in the biological processes category (FDR 1.1 × 10^−68^), while synapse (FDR 1.1 × 10^−69^) and enzyme binding (FDR 1.1 × 10^−31^) were overrepresented in cellular component and molecular function categories, respectively (Figure S6). Then, to identify a functional relationship between altered miRNAs and the dysregulated genes identified in contrasts A and B, we performed GO enrichment with the individual lists of putative targets per miRNA. This approach led us to identify 30 miR-222 dysregulated targets in MIO-M1-CTG_(648)_ Dox-treated cells, which were enriched in processes including cytokine-mediated signaling pathway (Table 4 and Figure 6). We determined 32 putative miR-103a-1 targets, involved in animal organ development and morphogenesis. miR-298 revealed to have 24 targets enriched in anatomical structure morphogenesis, among other biological processes. miR-4310 showed 18 targets, that were associated with Toll-like receptor signaling pathway and cytokine-mediated signaling pathway among other immune-related processes. We finally found miR-448 to have 24 putative target genes, involved in cellular response to cytokine stimulus and response to cytokine (Table 4 and Figure 6). Collectively, our results suggest that some of the observed gene changes in MIO-M1-CTG_(648)_ cells are caused by miRNA dysregulation.

## 4. Discussion

In this work we reported the generation of a new in vitro inducible model for DM1 based on MIO-M1 cells. These cells retain the functional and phenotypic features of Müller glia, including progenitor characteristics, electrophysiological response to glutamate and expression of the cell markers EGF-R, glutamate synthetase, and CRALB [31,48,49]; providing us an excellent biological tool to study the effects of the CTG expansion on glia physiology. The inducible DM1 MIO-M1 model established in this work recreated the RNA gain-of-function mechanism of the disease, exhibited global transcriptome changes, and, according to qPCR validation and GO enrichment analyses, presents inflammation pathway and immune response as major cellular impaired processes.

We demonstrated the stable incorporation of the transgene (Figure S1), and the specific expression of exogenous *DMPK* transcript in the inducible model (Figure 1A,B). Furthermore, by RNA-FISH experiments, we revealed the existence of abundant CUG RNA foci exclusively in the MIO-M1-CTG_(648)_ cells after Dox induction (Figure 1C). Notably, we observed dysregulation of the alternative splicing process of *MBNL1* and *MBNL2*, as well as co-localization of these splicing regulators with RNA foci in MIO-M1-CTG_(648)_ Dox-treated cells (Figure 2 and Figure S2). These results are in agreement with previous studies performed in cortical astrocytes of the DMSXL mouse and brain samples of affected humans [14,30]; demonstrating that in MIO-M1 DM1 cells, even lacking the *DMPK* genomic context, the CTG expansion is still able to recapitulate many molecular features of the disease, including splicing defects and accumulation of RNA foci colocalized with MBNL proteins. 

Identification of altered gene expression patterns in DM1, in both coding and non-coding genes, has gradually increased in the past few years [43,50,51]. This not only has increased complexity of the disease mechanism but has also raised the possibility of identifying biomarkers and design then novel therapeutic strategies [52]. Thus, we used our MIO-M1 DM1 cell model to explore effects of the DM1 mutation on global gene expression. By applying a microarray approach, we detected global transcriptome changes associated with the DM1 mutation (Figure 3). Comparative analysis revealed 316 and 790 differentially expressed genes in contrast A and contrast B, respectively. The difference in the total number of affected genes between both comparisons is likely due to a different insertion site of transgene in control [MIO-M1-CTG_(0)_] and mutant [MIO-M1-CTG_(648)_] cells. Despite of this fact, the proportion of up-regulated, down-regulated, coding and non-coding altered genes was fairly similar between these two contrasts (Figure 3). It is worth to note that a proportion of the most differentially events identified in both contrasts corresponded to the same genes, (see in Table 1: *CXCL10, TNFRSF9, CCL5, SCG2, MIR4288, PLEKHS1, TSPAN18*). More importantly, validation experiments of the selected transcripts by RT-qPCR supported the reliability of microarrays data (Table 2, and Figure 5). 

A major finding of this study is the abnormal transcript regulation of immune response mediators observed in the MIO-MI-CTG_(648)_ Dox-induced cells: the increased expression of *CXCL10 (*also known as *IP-10), TNFRSF9, TNFAIP3* (or *A20*)*, CXCL8 (IL-8),* and *CCL5* (Table 2), as well as the enrichment of important pathways including the immune response IL-1 signaling pathway (Table 3 and Figure 5). In concordance with our results, a previous microarray study, performed in lens epithelial samples of DM1 patients with cataract, reported 382 significant changes with enrichment in both interferon (IFN)-regulated genes and genes associated with the innate immune response [53]. More recently, participation of IFN1 pathway to rescue impaired differentiation of congenital DM1 myoblast was described [54]. Likewise, a study in a *Zebra fish* model expressing 91 CUG repeats, reported an upregulation of *Sesn3*, a marker of inflammation implicated in some neurological disorders [55]. Immune response in DM1 has also been explored at systemic level: higher levels of the soluble tumor necrosis factor receptor 2 (sTNFR2), interleukin (IL) 1 beta (IL-1β), tumor necrosis factor-alpha (TNF-α), and IL-6, as well as decreased levels of IL-10 have been found in the plasma of patients, compared to healthy subjects. Overall, these studies have revealed significant association between pro-inflammatory cytokines and both DM1 disease severity and CTG expansion size [56,57,58]. In the light of this, and in conjunction with our transcriptome data, an active role of the inflammation and immune response pathway in MIO-M1 cells is highly conceivable. 

A pro-inflammatory cellular environment may contribute to the derangement of CNS function [59]. Consistent with this notion, several studies have linked CNS pathology with altered levels of chemokines: CXCL10 has been implicated with altered cognitive function [60,61], and neuroendocrine dysregulation [62]; furthermore, altered levels of CXCL8 were found to be associated with schizophrenia [63], and depression [64], as well as with structural brain abnormalities [65]. Finally, dysregulation of CCL5 has been linked with depression and neuroinflammatory processes in neurodegenerative conditions, such as Alzheimer (AD) and Parkinson’s (PD) diseases [66]. Deregulated release of chemokines by glia cells may impact on neuron-glia communication through the orchestration of a neuroinflammatory cross-talk [67]. It has been reported that chemokines like CCL5, may lead to an activated glia state that releases pro-inflammatory effectors including TNF-α and IL-1 β, which in turn induce chemokine overproduction [68,69]. In line with this, we identified the TNF-α pathway, the immune response to IL-1 signaling, and the chemokine mediated signaling pathway as the most enriched key pathways and biological processes in MIO-M1-CTG_(648)_ cells (Table 3, GSEA and KPA analyses). In addition, we found overexpression of *TNFRSF9* (Table 2 and Table 3), a member of the TNF receptor family that activates microglia cells, enhances cell adhesion, and promotes secretion of pro-inflammatory cytokines [70,71]. We also observed upregulation of *TNFAIP3* (Table 2 and Table 3), a critical mediator of microglia activation and synaptic function [72]. In concordance with these results, a previous study showed increased microglial expression related to a proinflammatory process in the *Mbnl2* knockout mice, an animal model that displays features of DM1 neuropathophysiology [73]. Collectively, these observations support the existence of a neuroinflammatory mechanism in the Dox-induced MIO-M1 CTG_(648)_ cells. 

Interestingly, GSEA analysis identified enrichment of the splicing processes in MIO-M1-CTG_(648)_ Dox-induced cells (see spliceosomal complex for cellular component category in Table 3 and RNA splicing via transesterification reactions in Figure S3), which is in agreement with the alternative splicing defect at *MBNL1/2* exon 7 (Figure 2). Moreover, cell cycle progression (G2/M checkpoint) was found enriched in our analyses (Figure S3). In concordance with this, Peng et al. demonstrated that CUG expansion led to accelerated cell cycling, which explains at least in part, the defective myocyte differentiation in DM1 [74]. 

Remarkably, GO analyses uncovered the NFκB transcription factor as a potential key regulator of inflammation processes in MIO-M1-CTG_(648)_ cells (see TFT and PaPM categories in Table 3, and Figure 5). As reported elsewhere, NFκB exerts important functions in cell cycle and survival, immune response and inflammation [75,76,77,78,79]. After activation by myriads of agents (mitogens, viruses, oxidative stress, lipopolysaccharide, and cytokines, etc.), NFκB regulates the expression of multiple of genes including cytokines and chemokines. Solid experimental evidence has indicated that NFκB upregulates the expression of IRF1 [80], IL8 [46,81], CCL5 [81], and CXCL10 [47,82]. Thus, the upregulated expression of the immune mediators *CXCL10, CCL5, CCXCL8,* and *TNFRSF9* could be explained by increased activation of transcription regulators such as *RELB, NFκB2*, and/or *IRF1* (Figure 5), whose RNA expression was found affected in Dox-induced MIO-M1-CTG_(648)_ cells (Tables S2 and S3: LogFC 1.26/contrast A, LogFC 1.31/contrast B for *RELB*; LogFC 0.84/contrast A, LogFC 0.60/ contrast B for *NFκB2*; and LogFC 0.88/contrast A, LogFC 0.62/contrast B for *IRF1*). 

Several miRNAs have been found to be deregulated in tissues and cell models of DM1 [52,83,84,85]; however, studies in the CNS and particularly in glia cells are lacking. In this work, we detected dysregulated levels of six miRNAs in both in contrasts A and B (Table 4). None of these alterations, except for the miR-222 upregulation reported in skeletal muscle [86], has been previously reported in DM1. Functional consequences of these dysregulated miRNAs were evaluated by GO enrichment analysis of their respective targets. Such analysis revealed an involvement of the altered miRNAs in processes with relevance to CNS function, specifically in nervous system development (Figure S5). In line with this, miR-222 is associated with proliferation of neural stem cell, the development of retina [87], as well as with developing and maturation of the CNS [88]. On the other hand, the inhibition of miR-103a can inhibit the activation of astrocytes in hippocampus, improving thereby neuron damage [89]; furthermore, its low serum levels have been linked with cognitive impairment [90]. It is worth to note that dysregulated expression of miR-4288, miR-222, miR103, miR-298, and miR-448 found in our DM1 model is shared with other neurodegenerative conditions such as AD and Huntington’s disease (HD) [91,92,93,94,95]. Surprisingly, although miR-4288 showed the highest FC (2.79 contrast A/2.77 contrast B) in Dox-induced MIO-M1-CTG_(648)_ cells, we only recognized two targets within the dysregulated genes (Table 4). *CELF3* was identified as a miR-298 target, while *CELF5* and *CELF6* were both predicted as miR-448 targets. Interestingly, *MBNL1* was revealed as one of the predicted miR-4288 targets. Considering the central role playing by MBNL and CELF proteins in the DM1 pathogenic mechanism, further studies are required to explore the functional consequences of the indicated dysregulated miRNAs. 

As shown in Table 4, GO enrichment analysis of dysregulated miRNA targets suggested a miRNA-mediated regulation of the immune response in Dox-induce MIO-M1-CTG_(648)_ cells, mainly through, miR-222, miR-4310, and miR448 (Figure 6). Supporting this, it has been demonstrated that miR-222 directly activates NFκB [96], which in turn induce miR-222 expression [97], orchestrating a positive feedback loop. On the other hand, it has been reported the transcriptional suppression of miR-448 by NFκB [98]. These regulatory feedback loops may promote the increased expression of their predicted targets, namely, *TNFRSF9, CXCL8,* and *TNFAIP3* (Table 4), and ultimately induce the immune/inflammatory response in Dox-induced MIO-M1-CTG_(648)_ cells. The existence of multiple recognition sites for different miRNA on transcripts is well known [99]. Nonetheless, it is worth to note that miRNAs can also be regulated by target interactions in an intricate manner [100], meaning that transcript expression may be controlled at various levels.

Collectively, our findings strongly suggest the implication of the inflammatory pathway and the immune system response in the physiology of glial MIO-M1-CTG_(648)_ cells. The establishment of this glial DM1 cell model will be helpful to explore new mechanistic avenues that connect the glia with the CNS alterations of DM1 patients. Further functional experiments in MIO-M1-CTG_(648)_ cells as wells in animal models and/or brain human samples are required to strengthen the significance of our findings in the biology of DM1. 

## 5. Conclusions

We established an inducible glial cell model for DM1, the MIO-M1-CTG_(648)_ cells, which recapitulated the molecular hallmarks of the disease. The transcriptome alterations found in these cells open a new avenue for the study of glia as a contributor to the CNS symptoms of DM1, likely by the activation of a neuroinflammation processes throughout dysregulation of chemokines and immune meditators. Furthermore, our DM1 cell model might represent a valuable tool for the design of novel therapeutic approaches. 

## Figures and Tables

**Table 1:** List of the most up and down (-) differentially expressed genes in contrast A and B.

Contrast A			Contrast B		
Gene Symbol	Fold Change	*p* Value	Gene Symbol	Fold Change	*p* Value
*CXCL10*	4.66	4.49 × 10^−9^	*CXCL10*	9.19	6.24 × 10^−11^
*TNFRSF9*	4.63	2.51 × 10^−8^	*CCL5*	5.04	1.86 × 10^−7^
*CXCL8*	3.44	1.51 × 10^−6^	*SCG2*	4.32	3.26 × 10^−8^
*CCL5*	3.10	8.12 × 10^−6^	*TNFRSF9*	4.12	6.13 × 10^−8^
*SCG2*	3.10	7.30 × 10^−7^	*ATF3*	3.82	1.61 × 10^−7^
*PTX3*	2.79	1.47 × 10^−6^	*CXCL11*	3.35	1.21 × 10^−5^
*MIR4288*	2.78	1.43 × 10^−5^	*RFPL4AL1*	2.93	1.57 × 10^−3^
*BIRC3*	2.68	6.22 × 10^−6^	*PLA2G4C*	2.84	1.59 × 10^−7^
*PLA2G4C*	2.59	4.50 × 10^−7^	*LINC00707*	2.78	5.88 × 10^−6^
*HTR7P1*	2.58	2.53 × 10^−6^	*MIR4288*	2.76	1.53 × 10^−5^
*HHAT*	−1.56	6.74 × 10^−3^	*LINC00673*	−2.04	1.03 × 10^−6^
*SCD*	−1.66	9.01 × 10^−4^	*PLEKHS1*	−2.06	8.13 × 10^−6^
*PLXDC1*	−1.66	5.11 × 10^−5^	*LRMDA*	−2.07	1.47 × 10^−5^
*C3AR1*	−1.74	9.89 × 10^−5^	*PLXDC1*	−2.10	1.14 × 10^−6^
*KIF26B*	−1.76	3.89 × 10^−4^	*TSPAN18*	−2.12	1.36 × 10^−5^
*APLN*	−1.77	1.68 × 10^−4^	*SV2A*	−2.25	5.14 × 10^−7^
*SCG5*	−1.79	4.25 × 10^−5^	*APLN*	−2.28	4.76 × 10^−6^
*DCN*	−2.02	3.28 × 10^−3^	*COL11A1*	−2.59	8.67 × 10^−7^
*PLEKHS1*	−2.12	5.69 × 10^−6^	*KIF26B*	−2.74	1.63 × 10^−6^
*TSPAN18*	−2.27	5.60 × 10^−8^	*DCN*	−3.28	4.51 × 10^−5^

**Table 2:** Gene expression by RT-qPCR and microarray of selected genes.

Gene	Contrast A		Contrast B	
	FC RT-qPCR	FC MA	FC RT-qPCR	FC MA
*CXCL10*	25.2 (1.0 × 10^−4^)	4.66 (4.4 × 10^−9^)	228.2 (1.0 × 10^−4^)	9.19 (6.2 × 10^−11^)
*TNFRSF9*	11.7 (2.1 × 10^−3^)	4.63 (2.5 × 10^−8^)	10.2 (2.4 × 10^−3^)	4.12 (6.1 × 10^−8^)
*TNFAIP3*	4.1 (1.7 × 10^−2^)	2.53 (1.6 × 10^−7^)	2.2 (4.0 × 10^−2^)	1.97 (4.8 × 10^−6^)
*PLXDC1*	−5.8 (9.8 × 10^−3^)	−1.66 (5.1 × 10^−5^)	−2.4 (3.3 × 10^−2^)	−2.10 (1.1 × 10^−6^)
*TSPAN18*	−4.1 (1.0 × 10^−4^)	−2.27 (5.6 × 10^−6^)	−2.3 (2.3 × 10^−2^)	−2.12 (1.3 × 10^−5^)

FC = Fold change; MA = Microarray. Negative values mean down regulation. *p* values are showed in parenthesis.

**Table 3:** List of the most enriched ontology processes and pathways in MIO-M1CTG_(648)_ cells.

Resource	Category	Name	Genes		
				**Size**	**NES**
**GSEA**	H	TNF-α signaling pathway	*TNFRSF9, CXCL11, BRIC3, CCL5, PTX3, TNFAIP3, RELB, ATF3, IFIT2, IRF1, NFKB2*	131	2.43
	BP	Chemokine mediated signaling pathway	*CCR4, CXCL11, CCL5, CCL24, GPR75, MPL, CXCR3, CCL17, CCR6*	25	2.30
	CC	Spliceosomal complex	*PRPF39, SNRPB, SNRNP48, XAB2, PTBP2, ARSR2, LUC7L, BUD31, AQR, ZCCHC8*	137	1.91
	MF	Chemokine receptor binding	*CXCL10, CXCL11, CCL5*	24	2.02
	TFT	GGGNNTTTCC_NFκB_Q6_01	*CXCL10, TNFRSF9, BIRC3, CCL5, RELB, NFKB2, WNT10A, POUF2F3, CCND2*	85	2.09
	KP	Chemokine signaling pathway	*CCR4, CXCL10, CXCL11, CCL5, PLCB4, TIAM1HCK, ADCY4, PIK3R5, RAF1, PIK3CD*	118	1.89
				**%**	***p*** **val**
**DAVID**	BP	Inflammatory response	*CCL5, CXCL10, CCR4, CXCL11, CXCL8, RELB, TNFAIP3, TNFRSF9, C3AR1, IRGM, NFKB2, PTX3, PLA2G4C, SCG2*	12.1	2.9 × 10^−7^
	CC	Extracellular space	*CCL5, CXCL11, CXCL8, TNFRSF9, APLN, CST7, DCN, GHR, ITGAM, IFNA14, IL32, NRG2, PTX3, PLXDC1, SCG2, SERPIND1*	16.0	1.3 × 10^−3^
	MF	RNA Pol II regulatory region sequence-specific	*RELB, SOX13, ATF3, DLX2, NR1D1*	4.7	1.9 × 10^−2^
	KP	Cytokine-cytokine receptor interaction	*CCL5, CCR4, CXCL10, CXCL11, CXCL8, TNFRSF9, GHR, IFNA14, IL2RG*	7.5	7.9 × 10^−4^
**KPA**				**KH** ***p* val**	**Union** ***p* val**
	KPM	Immune response IL-1 signaling pathway	*CXCL8, CCL5, CXCL10, MAP3K14, MAPK14, JNK, IRAK1, RELA, IRF1, MAP3K8, NFKB2, CIAP2, IFNB.*	2.9 × 10^−7^	1.1 × 10^−12^
	PaPM	NF-kB pathway in multiple myeloma	*CIAP2, MAP3K14, IKKA, NFKB2, RELB, CYLD, IKB*	1.5 × 10^−4^	5.0 × 10^−9^
	PhPM	Immune response TNFR2 signaling pathways	*IKKA, RELA, TRAF1, IKB, NFKB, CIAP2, MAP3K14, IKKA, NFKB2, RELB, MAPK8-10, BCL-XL, C-JUN*	7.3 × 10^−7^	4.7 × 10^−11^

Categories: H = Hallmark, BP = Biological Process, CC = Cellular Component, MF = Molecular Function, TFT = Transcription Factor Targets, KP = KEGG Pathway, KPM = Key Pathway Maps, PaPM = Pathological Pathway Maps, PhPM = Physiological Pathway Maps. NES = Normalized Enrichment Score, KH = Key Hubs.

**Table 4:** Enriched ontology processes of predicted targets for dysregulated miRNAs in MIO-M1-CTG_(648)_ cells.

miRNA	FC	Target Genes	Biological Process	FDR
**miR-4288**	2.79	Up-regulated: *SYTL2, NRG2*	Not determined	-
**miR-222**	2.37	Up-regulated: *ATF3, BAMBI, BIRC3, CXCL11, IFIT2, IL2RG, KIF26B, KLHL1, LRRC49, PPP1R15A, PTX3, SERPIND1, SH3RF2, SNAPC1, SOX13, TUFA, TNFAIP3, TTC9, TUF1*	Cytokine-mediated signaling pathway Cytoplasmic pattern recognition receptor signaling pathway Regulation of molecular function Negative regulation of catalytic activity Intracellular signal transduction	1.8 × 10^−2^ 1.8 × 10^−2^ 1.8 × 10^−2^ 1.8 × 10^−2^ 1.9 × 10^−2^
		Down-regulated: *APLN, CD24, COL11A1, GHR, HHAT, PLEKHS1, PLXDC1, PSD3, SCG5, SV2A, SCD*		
**miR-103a1**	2.04	Up-regulated: *ATF3, CSRNP1, CYHR1, DLX2, DUSP4, HSPA4L, IRF1, MYLK3, PLA2G4C, PTX3, SERPIND1, SH3RF2, SYTL2, TIFA, TMEM45B, TNFAIP3, TTC9, TUFT1*	Animal organ development Animal organ morphogenesis Skeletal system morphogenesis Skeletal system development Anatomical structure morphogenesis Regulation of signal transduction	1.3 × 10^−3^ 3.9 × 10^−3^ 4.0 × 10^−3^ 1.3 × 10^−2^ 1.6 × 10^−2^ 1.9 × 10^−2^
		Down-regulated: *APLN, CD24, COL11A1, DCN, EYA1, GHR, HHAT, KIF26B, PLEKHS1, PLXDC1, PSD3, SCD, SV2A, TSPAN18*		
**miR-298**	1.91	Up-regulated: *BAMBI, CCL5, CSRNP1, DUSP4, HSPA4L, KLHL1, MYLK3, NR1D1, PLCB4, SCG2, SNAPC1, SOX13, SYTL5, TNFAIP3, TUFT1*	Anatomical structure morphogenesis Negative regulation of signal transduction Regulation of cell communication Regulation of signaling Regulation of chronic inflammatory response	2.8 × 10^−3^ 2.8 × 10^−3^ 2.8 × 10^−3^ 2.8 × 10^−3^ 2.8 × 10^−3^
		Down-regulated: *APLN, CD24, DCN, GHR, PLXDC1, PSD3, SCD, SCG5, TSPAN18*		
**miR-4310**	1.48	Up-regulated: *ATF3, BIRC3, IFIT2, IRF1, ITGAM, KCTD16, PTX3, SNAPC1, SYTL5, TNFAIP3, TTC9*	Toll-like receptor signaling pathway Cytokine-mediated signaling pathway Activation of innate immune response Cellular response to cytokine stimulus	2.7 × 10^−3^ 4.9 × 10^−3^ 5.5 × 10^−3^ 8.4 × 10^−3^
		Down-regulated: *APLN, CD24, PLEKHS1, PLXDC1, PSD3, SCD, SV2A*		
**miR-448**	−1.43	Up-regulated: *CXCL8, DHRS2, HSPA4L, IFIT2, IRF1 TNFRSF9, KCTD16, LRRC49, MYLK3, NRG2, PLCB4, SGIP1, SH3RF2, SNAPC1, SOX13, TNFAIP3, TTC9, TUFT1, ZNF620*	Cellular response to cytokine stimulus Response to cytokine Cytokine-mediate signaling pathway Defense response to virus	2.0 × 10^−2^ 2.0 × 10^−2^ 2.0 × 10^−2^ 2.8 × 10^−2^
		Down-regulated: *EYA1, GHR, PSD, SCD3, SCG5*		

FC = Fold change. Negative value (-) mean down regulation.

## Data Availability

The microarray data of this study are available in NCBI’s Gene Expression Omnibus database (GSE164057).

## Supplementary Materials

The following are available online at https://www.mdpi.com/2218-273X/11/2/159/s1. Figure S1: Generation of cell model and genotyping. Figure S2: *MBNL1/2* splicing defects and MBNL1 colocalization with RNA foci. Figure S3: RT-qPCR validation of microarray gene expression analysis. Figure S4: GSEA of non-immune related enriched pathways in Dox-induced MIO-M1-CTG_(648)_ cells. Figure S5: Immune response IL-1 signaling pathway regulates numerous cell functions potentially altered in Dox-induced MIO-M1-CTG_(648)_ cells. Figure S6: GO enrichment analysis of dysregulated miRNA targets, filtered by brain tissue. Table S1: Sequences of primers used in RT-PCR assays. Table S2: Overlapping dysregulated genes detected in contrast A. Table S3: Overlapping dysregulated genes detected in contrast B.
