# Supplementary material for: Transcriptome Analysis Reveals Altered Inflammatory Pathway in an Inducible Glial Cell Model of Myotonic Dystrophy Type 1
Source: Biomolecules. 2021 Jan 26;11(2):159. doi: 10.3390/biom11020159 (PMC7910866; doi:10.3390/biom11020159)
Supplement: Supplementary file 1 [file biomolecules-11-00159-s001.pdf]

biomolecules

Supplementary Figures and Tables

Transcriptome analysis reveals altered inflammatory pathway in an inducible glial cell model of myotonic dystrophy type 1

Cuauhtli N. Azotla-Vilchis [1,2], Daniel Sanchez-Celis [1,2], Luis E. Agonizantes-Juárez [1,3], Rocío Suárez-Sánchez [1], J. Manuel Hernández-Hernández [2], Jorge Peña [4,5], Karla Vázquez-Santillán [6], Norberto Leyva-García [1], Arturo Ortega [7], Vilma Maldonado [6], Claudia Rangel [4], Jonathan J. Magaña [1,8], Bulmaro Cisneros [2] and Oscar Hernández-Hernández [1,*]

[1] Laboratory of Genomic Medicine, Department of Genetics, Instituto Nacional de Rehabilitación, Luis Guillermo Ibarra Ibarra , Mexico City 14389, Mexico; cuauhtli_azotla@yahoo.com.mx (C.N.A.-V.); ny3687@hotmail.com (D.S.-C.); gen.bioq@gmail.com (L.E.A.-J.); srossmary@gmail.com (R.S.-S.); nleyga06@gmail.com (N.L.-G.); maganasm@hotmail.com (J.J.M.)

[2] Department of Genetics and Molecular Biology, Centro de Investigación y de Estudios Avanzados, CINVESTAV-IPN. Mexico City 07360, Mexico; jose.hernandezh@cinvestav.mx (J.M.H.-H.); bcisnero@cinvestav.mx (B.C.)

[3] Escuela Nacional de Ciencias Biologicas-Instituto Politécnico Nacional, Mexico City 11340, Mexico

[4] Computational and Integrative Genomics Laboratory. Instituto Nacional de Medicina Genómica, Mexico City 14610, Mexico; crangel@inmegen.gob.mx (C.R.); jorge.pena@cgu.edu (J.P.)

[5] Institute of Mathematical Sciences, Claremont Graduate University, Claremont CA 91711, USA

[6] Epigenetics Laboratory, Instituto Nacional de Medicina Genomica, Mexico City 14610, Mexico; kivs09@gmail.com (K.V.-S); vilmaml@gmail.com (V.M.)

[7] Department of Toxicology, Centro de Investigación y de Estudios Avanzados, CINVESTAV-IPN, Mexico City 07360, Mexico; arortega@cinvestav.mx

[8] School of Engineering and Sciences. Department of Bioengineering. Tecnológico de Monterrey-Campus Mexico City 14380, Mexico

* Correspondence: heroscar@gmail.com or ohernandez@inr.gob.mx; Tel: +52-55-5999-1000 (ext. 14710)

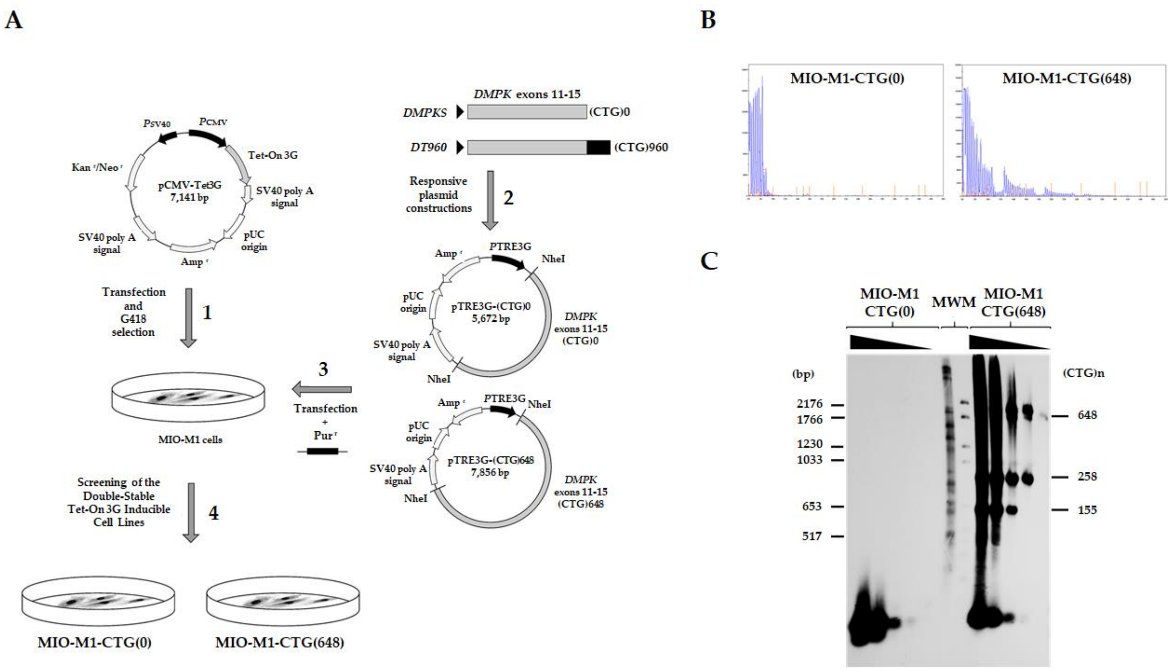

Figure S1. Generation of cell model and genotyping. (**A**). Regulatory pCMV-Tet3G plasmid encoding trans activator was stably transfected into MIO-M1 cells which were subjected to G418 selection (1), In parallel, *DMPK* minigenes carrying 960 or 0 CTG repeats, were cloned into the NheI site of pTRE3G responsive plasmid (2). Each responsive plasmid was transfected into the MIO-M1 cells expressing trans activator and cultured under the presence of puromycin (3). After expansion and screening (4) MIO-M1-CTG$_{(0)}$ and MIO-M1-CTG$_{(648)}$ were established. (**B**) Electropherograms of PCR products for amplifications of the CTG repeat region of the *DMPK* gene using DNA from MIO-M1-CTG$_{(0)}$ and MIO-M1-CTG$_{(648)}$ cells. (**C**) SP-PCR performed with serial dilutions of DNA obtained from MIO-M1-CTG$_{(0)}$ and MIO-M1-CTG$_{(648)}$ cells. Signal at the bottom of gels corresponds to normal alleles. PCR products carrying CTG expansion, corresponding with a 648 CTG tract, were detected only in MIO-M1-CTG$_{(648)}$ cells.

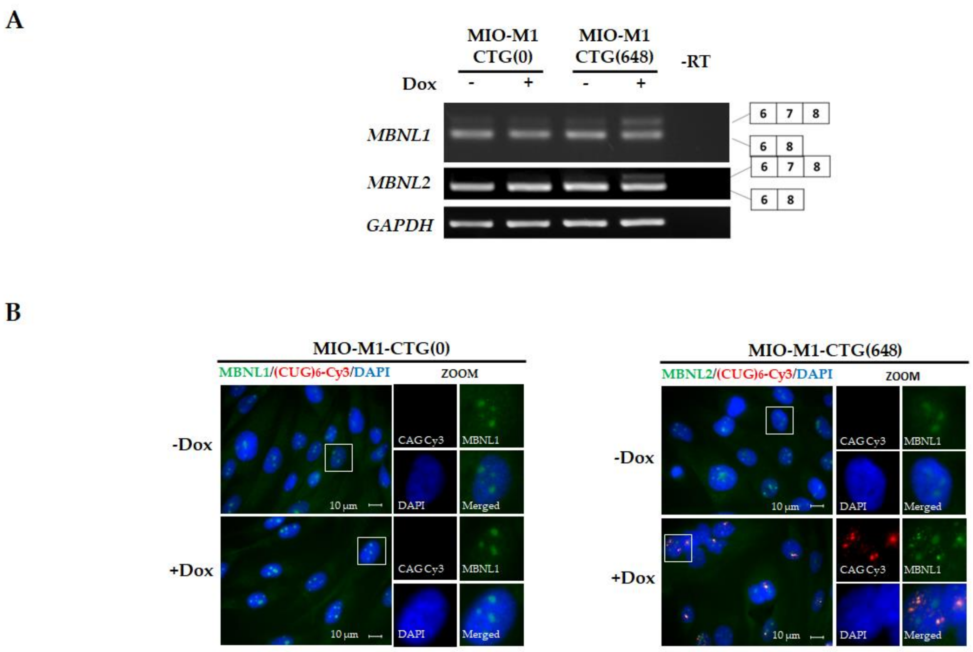

Figure S2: *MBNL1/2* splicing defects and MBNL1 colocalization with RNA foci. (A) Representative RT-PCR splicing analysis of MBNL1 and MBNL2 exon 7 in MIO-M1-CTG$_{(0)}$ and MIO-M1-CTG$_{(648)}$ cells before (-) and after (+) dox induction. (**B**) RNA FISH [(CAG)6- Cy3] and immunofluorescence (MBNL1) showed colocalization of mutant RNA with MBNL1 exclusively in MIO-M1-CTG$_{(648)}$ upon dox induction (+Dox). Cells were counterstained with DAPI for nuclei visualization prior to being analyzed by confocal microscopy. 4.5X magnification (ZOOM) is showed for each condition.

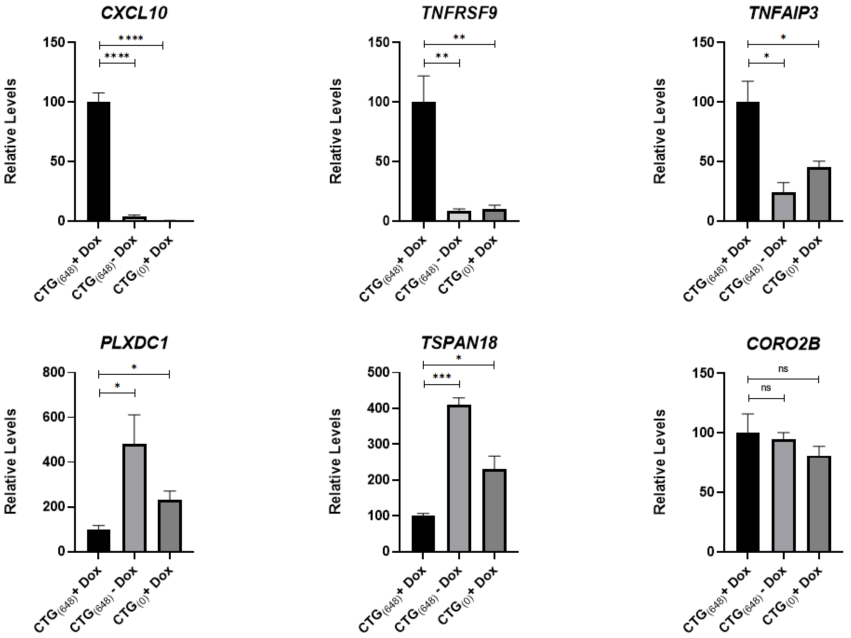

Figure S3. RT-qPCR validation of microarray gene expression analysis. Graphs are presented as the mean ± SEM of normalized expression values of three independent experiments, with p values indicating significance differences (unpaired t-test), *denotes p>0.05, **denotes p<0.005, ***denotes p<0.0005. n.s. not significant.

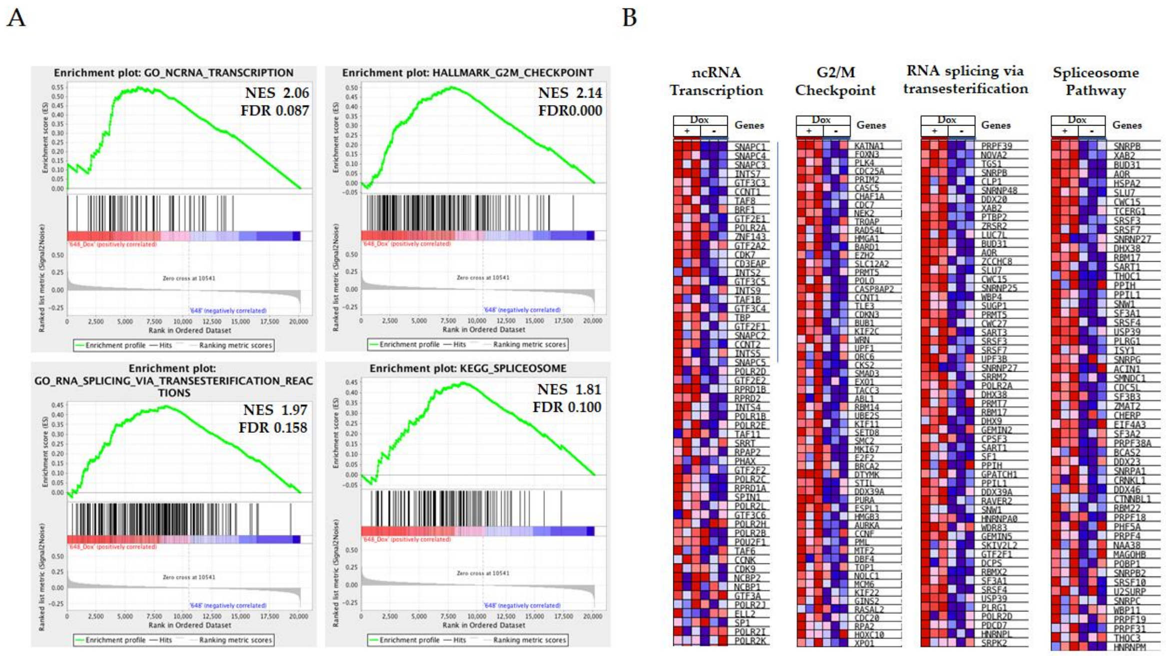

Figure S4. GSEA of non-immune related enriched pathways in Dox-induced MIO-M1-CTG(648) cells. Enrichment plots of four highly significant enriched pathways are showed. Leading edge subset denote the more biologically important genes, which are showed in the corresponding heat map for Dox-induced MIO-M1 CTG(648) cells (+ Dox) and non-induced MIO-M1 CTG(648) cells (- Dox) as control. Red: up-regulated, blue: down-regulated. Normalized enrichment score (NES) and false discovery rate (FDR) are indicated.

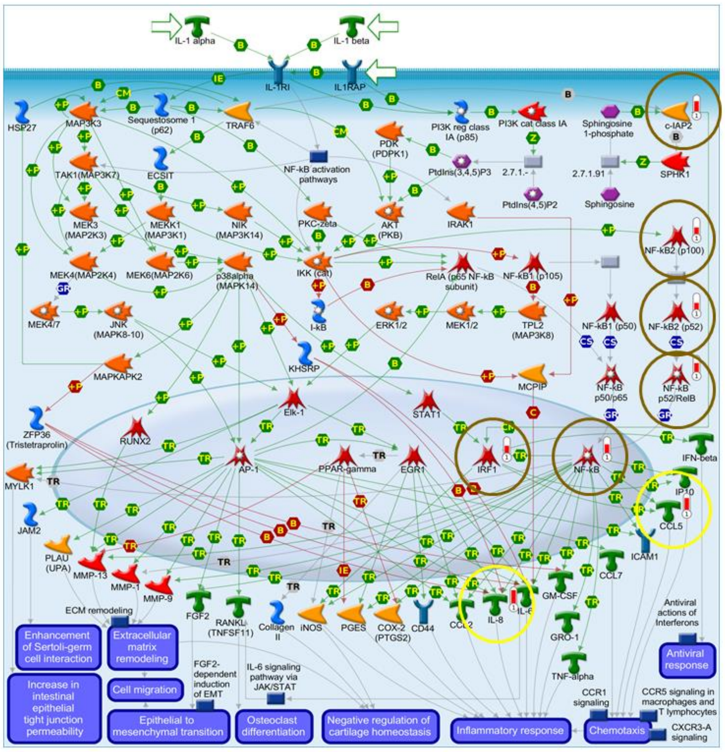

Figure S5. Immune response IL-1 signaling pathway regulates numerous cell functions (blue rectangles) potentially altered in MIO-M1-CTG(648) cells. Altered genes detected by microarrays are highlighted in brown circles. Some of the RT-qPCR validated targets of this pathway (*CXCL10, CCL5* and *CXCL8*), are indicated in yellow circles.

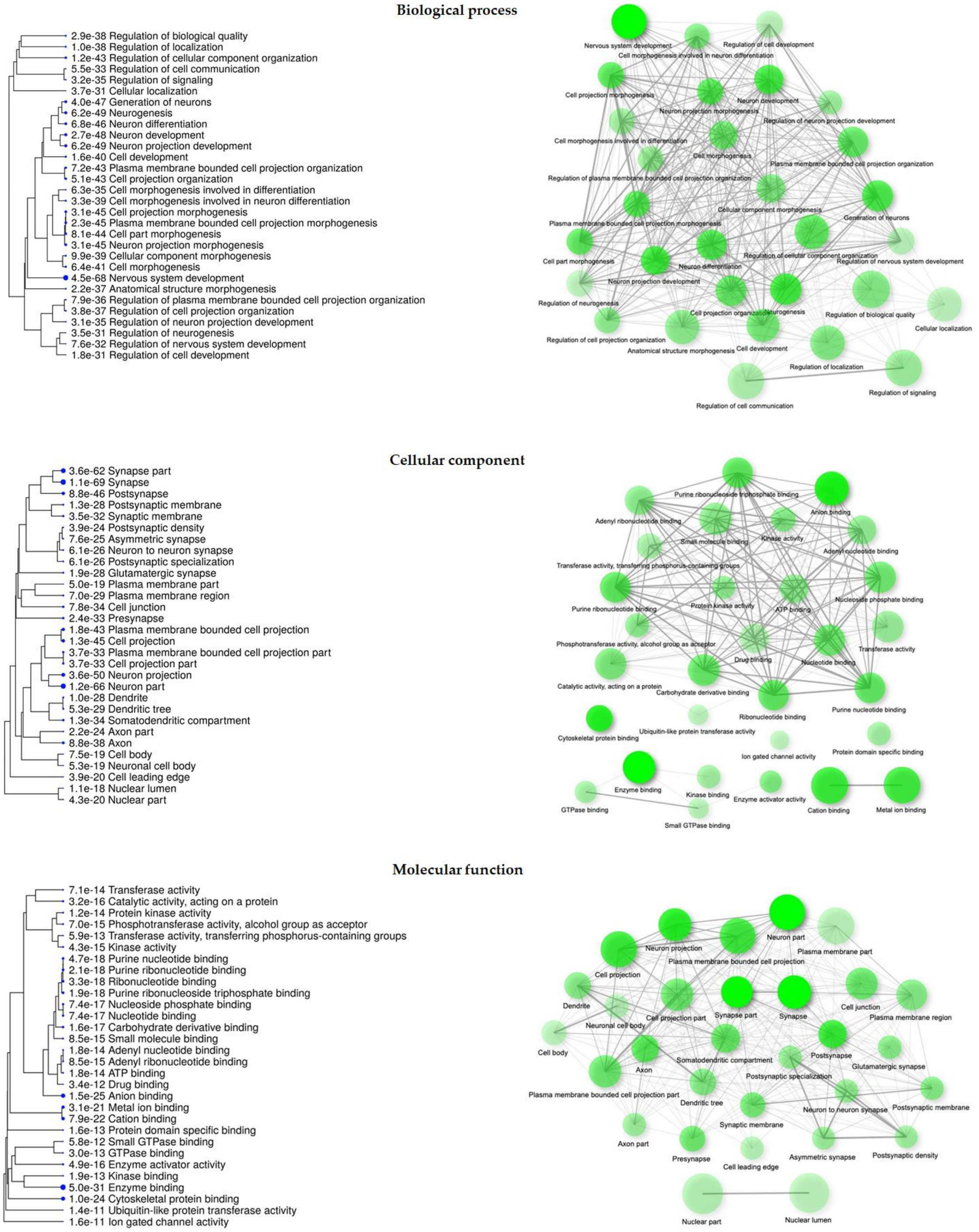

Figure S6. GO enrichment analysis of dysregulated miRNA targets, filtered by nervous system tissue. Enriched processes in MIO-M1-CTG(648) Dox-induced cells, for the indicated category are showed. Hierarchical clustering trees (left), and network diagrams (right) were performed using ShinyGO server. Nodes (circles) on networks, denote enriched processes; lines denote interactions among genes that are common between processes.

Table S1. Sequences of primers used in RT-PCR assays

Gene	Primer	Sequence (5´-3´)	PCR annealing temperature (°C)
GAPDH	GAPDH F<br>GAPDH R	TGATGACATCAAGAAGGTGGTGAA<br>TCCTTGGAGGCCATGTGGGCCAT	64
DMPK	pTRE3G F<br>DMPK R	GTCAGATCGCCTGGAGCAAT<br>GACGTGTGCCTCTAGGTCCC	67
MBNL2	MBNL F<br>MBNL R	ACAAGTGACAACACCGTAACCG<br>TTTGGTAAAGGATGAAGAGCACC	60
MBNL1	MBNL F<br>MBNLR	GCTGCCCAATACCAGGTCAAC<br>TGGTGGGAGAAATGCTGTATGC	62

Table S2. Overlapping dysregulated genes detected in contrast A

Affymetrix ID	Gene Symbol	FC	LogFC	Ave Expr	t	P. val
TC0400011053.hg.1	CXCL10	4.65892842	2.21999816	5.07656105	14.6070401	4.49394E-09
TC0100012734.hg.1	TNFRSF9	4.63380729	2.21219805	7.98467492	12.5704582	2.50778E-08
TC0400007836.hg.1	CXCL8	3.43600154	1.78073068	8.26350365	8.65950777	1.51217E-06
TC1700010447.hg.1	CCL5	3.10194571	1.63317344	8.72249041	7.35991188	8.12129E-06
TC0200015865.hg.1	SCG2	3.01743119	1.59332087	7.58934046	9.27170716	7.29753E-07
TC0300009301.hg.1	PTX3	2.78856331	1.47952203	8.65423887	8.68390781	1.46782E-06
TC0800009986.hg.1	MIR4288	2.78262503	1.47644651	7.35043006	6.95658835	1.42544E-05
TC1100013087.hg.1	BIRC3	2.68341393	1.42406962	7.23148928	7.55611906	6.22221E-06
TC1900011057.hg.1	PLA2G4C	2.58541754	1.37039729	6.49910325	9.69566829	4.50123E-07
TC1200006904.hg.1	HTR7P1	2.58049125	1.36764574	9.80280192	8.24565223	2.52981E-06
TC0600009597.hg.1	TNFAIP3	2.53017375	1.33923646	9.67589146	10.637569	1.62932E-07
TC1900008932.hg.1	RFPL4AL1	2.50831526	1.32671869	4.75929016	3.46954345	0.004553156
TC0300006985.hg.1	CCR4	2.45784265	1.29739256	4.81206578	5.43741813	0.000144079
TC0400011054.hg.1	CXCL11	2.39301922	1.25883199	5.31811611	5.10671388	0.000248746
TC1900008300.hg.1	RELB	2.38960015	1.25676923	8.85083283	9.79744954	4.01817E-07
TC0X00011361.hg.1	MIR222	2.38042311	1.25121803	5.36548661	3.69855185	0.002981789
TC1400010683.hg.1	SNORD114-24	2.13008273	1.09090946	3.79652162	4.82968993	0.000397625
TC0100011533.hg.1	ATF3	2.10196198	1.07173657	7.9503571	5.90412678	6.84315E-05
TC2000006674.hg.1	PLCB4	2.06491022	1.04607906	5.72942027	5.63142857	0.000105335
TC0700009230.hg.1	AKR1B10	2.06222098	1.04419893	5.26910789	4.34986091	0.000917857
TC0X00006966.hg.1	SYTL5	2.06041057	1.04293185	6.64818514	5.90479022	6.83606E-05
TC1600006658.hg.1	IL32	2.05632053	1.04006516	8.34583038	6.30906191	3.67625E-05
TC0500012757.hg.1	MIR103A1	2.0430626	1.03073341	5.02243068	3.95524377	0.001864562
TC1000008396.hg.1	IFIT2	2.0418976	1.02991052	9.4307925	8.32906163	2.27718E-06
TC0800007389.hg.1	TCIM	1.9861924	0.99000538	6.79410514	5.79426918	8.13155E-05
TC0X00009981.hg.1	IL2RG	1.97506873	0.98190286	5.69996901	4.40809229	0.000827965
TC1400010620.hg.1	SNAPC1	1.9703571	0.97845712	9.39386546	7.97222698	3.59111E-06
TC0500009103.hg.1	IRGM	1.96237712	0.97260232	4.28464206	3.95682842	0.001859201
TC1500007735.hg.1	LRRC49	1.94168657	0.95731034	8.80533673	6.0419785	5.52439E-05
TC0X00006476.hg.1	CD99P1	1.93367248	0.95134345	8.91710456	5.59688562	0.000111333
TC1600011440.hg.1	OSGIN1	1.9186718	0.94010795	8.85696067	8.88269084	1.15436E-06
TC0600011798.hg.1	TFEB	1.90892082	0.93275727	5.69019787	5.3723522	0.000160227

TC2000009639.hg.1	MIR298	1.90640393	0.93085383	6.7537373	5.09416863	0.000254028
TC1400010594.hg.1	LOC101927178	1.89781302	0.92433786	4.33191974	6.49801969	2.77219E-05
TC1700010604.hg.1	NR1D1	1.88175089	0.91207565	11.0960379	7.82515711	4.35104E-06
TC0500009357.hg.1	MIR103B1	1.87946279	0.91032035	4.05113064	6.65213912	2.20999E-05
TC0700009231.hg.1	AKR1B15	1.87667332	0.90817753	6.91852267	5.39507618	0.000154381
TC0500012017.hg.1	IRF1	1.84416025	0.88296403	9.09677426	6.12257405	4.88044E-05
TC1000006683.hg.1	LINC00707	1.80554167	0.85243172	7.42258462	4.39720328	0.000844051
TC1000008727.hg.1	NFKB2	1.7866014	0.83721779	9.98442603	7.38417999	7.85609E-06
TC0100008664.hg.1	GADD45A	1.76760689	0.82179746	10.7701069	7.58190993	6.01021E-06
TC0X00006835.hg.1	MAGEB18	1.766908	0.82122692	3.64297928	4.09334295	0.001452168
TC1300009966.hg.1	LOC647264	1.76609634	0.82056404	3.94917726	5.53090913	0.000123813
TC2000008099.hg.1	UCKL1-AS1	1.76445888	0.81922581	6.75138442	3.61096178	0.00350431
TC0400011616.hg.1	TIFA	1.76112906	0.81650064	7.59650026	4.65276556	0.000539426
TC0400008785.hg.1	NOCT	1.72339723	0.78525527	10.0042028	5.45496891	0.000140024
TC1400006697.hg.1	DHRS2	1.70933198	0.77343262	6.4397961	4.97382996	0.000311105
TC0500013251.hg.1	KCTD16	1.65008479	0.72254016	4.25984422	3.69673419	0.00299178
TC1000008400.hg.1	IFIT1	1.64961085	0.72212573	9.11539383	3.55375155	0.003895253
TC0200009376.hg.1	GPR39	1.63764543	0.71162303	8.07921316	4.28225991	0.001035053
TC0800012216.hg.1	CYHR1	1.63322131	0.7077203	12.2411329	6.13661676	4.77662E-05
TC2200006675.hg.1	SERPIND1	1.61565427	0.69211852	4.72131025	4.09544411	0.001446678
TC1100009514.hg.1	TMEM45B	1.60871596	0.68590962	5.09520063	4.12339281	0.001375658
TC0500012784.hg.1	LINC01187	1.5946213	0.67321384	4.62468148	4.75243736	0.000454035
TC0300007123.hg.1	ZNF620	1.59411759	0.67275805	5.27981912	3.80168217	0.002467461
TC0100011282.hg.1	SOX13	1.58289562	0.66256613	10.3869961	6.13814579	4.76545E-05
TC1700009670.hg.1	ALOXE3	1.57501073	0.65536165	6.51620873	3.93520595	0.001933742
TC0200014912.hg.1	DLX2	1.57188387	0.65249463	8.36939349	4.67248247	0.000521293
TC1100011182.hg.1	PYGM	1.56504492	0.64620407	5.01849805	4.06879227	0.001517935
TC1100011833.hg.1	SYTL2	1.55882249	0.64045665	9.78374147	5.52237449	0.000125531
TC1200010776.hg.1	KRT4	1.55358382	0.63560008	5.08848287	4.96563882	0.00031545
TC0100009959.hg.1	TUFT1	1.55095906	0.63316061	11.0108075	5.41703959	0.000148944
TC1200010341.hg.1	PKP2	1.53577194	0.61896399	9.29322269	4.47297008	0.000738502
TC1000007132.hg.1	BAMBI	1.5268341	0.61054331	9.1059875	3.79546512	0.002495721
TC0300010770.hg.1	CSRNP1	1.52279938	0.60672589	7.66888812	4.84065173	0.000390236
TC0800010002.hg.1	DUSP4	1.52179895	0.60577777	8.31176849	5.33554503	0.000170196
TC0100016901.hg.1	CACNA1S	1.52088864	0.60491452	5.53220612	3.7924756	0.002509428
TC1600010174.hg.1	MYLK3	1.51100055	0.59550419	6.49741087	3.53254669	0.004051211
TC1900006652.hg.1	TJP3	1.50839104	0.59301049	5.17503064	3.435988	0.00484586
TC1300009204.hg.1	KLHL1	1.50746064	0.59212033	4.6065569	3.79547736	0.002495665
TC0600014102.hg.1	HCP5	1.49620073	0.58130374	7.7894498	5.45214454	0.000140668
TC1400010041.hg.1	IFI27L2	1.4858218	0.5712611	11.2270543	5.47432002	0.000135691
TC1500009172.hg.1	MIR4310	1.48176774	0.56731933	4.21323151	3.88318162	0.002125961
TC1600007528.hg.1	ITGAM	1.47771693	0.56336994	5.71621081	3.56352736	0.003825427
TC0500012223.hg.1	NRG2	1.47655748	0.56223752	8.43223457	4.22399818	0.001148489
TC1900011882.hg.1	ZNF442	1.46875815	0.55459686	7.97204819	3.96646806	0.00182693
TC0100008631.hg.1	SGIP1	1.46656911	0.55244506	8.1607322	3.4912434	0.004373498
TC0400008663.hg.1	HSPA4L	1.46520264	0.55110021	9.87371136	4.84954907	0.000384343
TC1400007584.hg.1	TTC9	1.46097486	0.54693136	5.09815657	3.49067608	0.004378102
TC0900009700.hg.1	IFNA14	1.4604674	0.54643015	6.40651451	3.85075858	0.002255597
TC0Y00006474.hg.1	CD99P1	1.45613443	0.54214355	8.26185847	3.58677435	0.003664462

TC1900008496.hg.1	PPP1R15A	1.45450942	0.54053264	10.3916016	4.68764097	0.000507785
TC1000008619.hg.1	NKX2-3	1.44370697	0.52977795	6.79428231	3.9993223	0.001721211
TC0500008994.hg.1	SH3RF2	1.43444795	0.52049562	7.83859088	3.42903131	0.004908897
TC1700012332.hg.1	SPATA22	1.42393777	0.50988609	4.41158956	3.5658777	0.003808831
TC0400008106.hg.1	HERC5	1.42303157	0.50896766	9.19983697	3.73384478	0.002794437
TC0800010774.hg.1	EYA1	-1.4153179	-0.5011262	11.4879738	-4.2582713	0.001080283
TC1500009830.hg.1	LCTL	-1.4219254	-0.5078458	8.51683153	-4.4215818	0.000808477
TC0600012747.hg.1	CD24	-1.423738	-0.5096837	11.1415955	-4.7306856	0.000471383
TC1200007690.hg.1	TNS2	-1.4261742	-0.5121502	8.13487177	-4.1288089	0.001362317
TC0100015714.hg.1	SV2A	-1.4273364	-0.5133254	11.0206927	-4.1950706	0.001209523
TC0100006873.hg.1	RNU5E-1	-1.4285248	-0.514526	9.62121242	-3.7420624	0.002752571
TC0X00008188.hg.1	MIR448	-1.4315838	-0.5176121	4.55658969	-3.9448238	0.001900213
TC1700012466.hg.1	LINC00673	-1.4436026	-0.5296736	8.72043514	-4.6265729	0.000564538
TC0800009764.hg.1	PSD3	-1.498719	-0.5837299	9.04197934	-3.3932683	0.00524634
TC0100015102.hg.1	COL11A1	-1.5157476	-0.6000295	11.2614544	-3.9803309	0.001781528
TC1700010590.hg.1	GSDMB	-1.524881	-0.6086967	6.87474711	-3.6694601	0.003145865
TC1000008099.hg.1	LRMDA	-1.5341945	-0.6174814	8.44997039	-4.0707771	0.001512505
TC0800008140.hg.1	ATP6V0D2	-1.5364293	-0.6195814	8.16892952	-3.7931676	0.002506249
TC0500007258.hg.1	GHR	-1.5545042	-0.6364545	8.74000979	-4.0851676	0.001473735
TC2000007005.hg.1	CST7	-1.5585135	-0.6401706	8.18023389	-5.4047958	0.00015195
TC0100011472.hg.1	HHAT	-1.5646069	-0.6458002	8.07158497	-4.5247494	0.000674343
TC1000008643.hg.1	SCD	-1.6596059	-0.7308407	12.373124	-4.3603767	0.000900903
TC1700010560.hg.1	PLXDC1	-1.6622665	-0.7331517	9.20252491	-6.0923191	5.11233E-05
TC1200009807.hg.1	C3AR1	-1.7439419	-0.802352	6.39722751	-5.670713	9.89257E-05
TC0100012247.hg.1	KIF26B	-1.7647426	-0.8194578	8.82455013	-4.8428367	0.00038878
TC0X00010787.hg.1	APLN	-1.7656004	-0.8201588	10.212155	-5.341827	0.000168449
TC1500006786.hg.1	SCG5	-1.7878398	-0.8382175	8.96346216	-6.2127968	4.25271E-05
TC1200011496.hg.1	DCN	-2.0173298	-1.012447	8.23121539	-3.6462296	0.003283497
TC1000008955.hg.1	PLEKHS1	-2.1158464	-1.0812349	8.75069761	-7.6226099	5.69118E-06
TC1100007400.hg.1	TSPAN18	-2.2703092	-1.1828888	9.5276138	-7.6344707	5.60165E-06

Table S3. Overlapping dysregulated genes detected in contrast B

Affymetrix ID	Gene Symbol	FC	LogFC	Ave Expr	t	P. val
TC0400011053.hg.1	CXCL10	9.18958684	3.19835965	5.07656105	21.0444173	6.23529E-11
TC1700010447.hg.1	CCL5	5.03611681	2.33231175	8.72249041	10.5105854	1.86043E-07
TC0200015865.hg.1	SCG2	4.31980949	2.11096769	7.58934046	12.2839502	3.25632E-08
TC0100012734.hg.1	TNFRSF9	4.12325632	2.04378415	7.98467492	11.6134734	6.12837E-08
TC0100011533.hg.1	ATF3	3.81768506	1.93269809	7.9503571	10.6471076	1.61325E-07
TC0400011054.hg.1	CXCL11	3.34718817	1.74294966	5.31811611	7.07063794	1.21326E-05
TC1900008932.hg.1	RFPL4AL1	2.92509324	1.54848261	4.75929016	4.04948522	0.001571811
TC1900011057.hg.1	PLA2G4C	2.84190045	1.50685602	6.49910325	10.6611245	1.58995E-07
TC1000006683.hg.1	LINC00707	2.77594416	1.47297855	7.42258462	7.59824624	5.87992E-06
TC0800009986.hg.1	MIR4288	2.76157041	1.46548891	7.35043006	6.9049593	1.53417E-05
TC0300006985.hg.1	CCR4	2.65008098	1.40603644	4.81206578	5.8927485	6.96597E-05

TC2000006674.hg.1	PLCB4	2.62097886	1.39010572	5.72942027	7.48345071	6.86359E-06
TC0X00011361.hg.1	MIR222	2.52891936	1.33852104	5.36548661	3.95661613	0.001859919
TC1200006904.hg.1	HTR7P1	2.5027949	1.32354007	9.80280192	7.9797354	3.55633E-06
TC0800007389.hg.1	TCIM	2.49782354	1.32067156	6.79410514	7.72958075	4.93578E-06
TC0400007836.hg.1	CXCL8	2.48568641	1.3136443	8.26350365	6.38811536	3.26486E-05
TC1900008300.hg.1	RELB	2.47811799	1.30924488	8.85083283	10.2065362	2.56972E-07
TC0X00006966.hg.1	SYTL5	2.45502412	1.2957372	6.64818514	7.33610387	8.39076E-06
TC1600006658.hg.1	IL32	2.40478477	1.26590778	8.34583038	7.67902898	5.27848E-06
TC0100008631.hg.1	SGIP1	2.3926862	1.2586312	8.1607322	7.95407221	3.67672E-06
TC0500012757.hg.1	MIR103A1	2.39197002	1.25819931	5.02243068	4.82810097	0.000398709
TC0800010002.hg.1	DUSP4	2.36690699	1.24300302	8.31176849	10.9480718	1.18439E-07
TC1100013087.hg.1	BIRC3	2.36638379	1.24268407	7.23148928	6.59368665	2.40747E-05
TC1500007735.hg.1	LRRC49	2.32865159	1.2194948	8.80533673	7.69673225	5.15566E-06
TC0700009231.hg.1	AKR1B15	2.31056564	1.20824607	6.91852267	7.17764906	1.04456E-05
TC1000008400.hg.1	IFIT1	2.23579739	1.16078945	9.11539383	5.71251951	9.25547E-05
TC0700009230.hg.1	AKR1B10	2.2145218	1.1469952	5.26910789	4.7780834	0.000434434
TC1600011440.hg.1	OSGIN1	2.15776256	1.10953612	8.85696067	10.4835474	1.91404E-07
TC0X00006476.hg.1	CD99P1	2.15637583	1.10860865	8.91710456	6.52209857	2.67518E-05
TC0600011798.hg.1	TFEB	2.14150493	1.098625	5.69019787	6.3276917	3.57458E-05
TC1400010683.hg.1	SNORD114-24	2.13304188	1.09291229	3.79652162	4.83855689	0.000391637
TC1400006697.hg.1	DHRS2	2.11949767	1.08372238	6.4397961	6.96925716	1.40003E-05
TC0X00009981.hg.1	IL2RG	2.08233408	1.05820155	5.69996901	4.75062278	0.000455456
TC1300009966.hg.1	LOC647264	2.06775694	1.04806661	3.94917726	7.06436169	1.22401E-05
TC0600009597.hg.1	TNFAIP3	1.96679369	0.97584563	9.67589146	7.75115189	4.79684E-06
TC0300007123.hg.1	ZNF620	1.94136092	0.95706836	5.27981912	5.40828859	0.000151086
TC0500009103.hg.1	IRGM	1.92054294	0.94151422	4.28464206	3.83035304	0.002341322
TC1400010594.hg.1	LOC101927178	1.91807245	0.93965721	4.33191974	6.60571349	2.36535E-05
TC0500013251.hg.1	KCTD16	1.89930526	0.9254718	4.25984422	4.73499387	0.000467893
TC2200006675.hg.1	SERPIND1	1.87891117	0.90989686	4.72131025	5.38409487	0.000157178
TC2000009639.hg.1	MIR298	1.86898483	0.90225486	6.7537373	4.93765856	0.000330777
TC0100008664.hg.1	GADD45A	1.86526498	0.8993806	10.7701069	8.29769258	2.36887E-06
TC1000008396.hg.1	IFIT2	1.84943232	0.8870825	9.4307925	7.17398718	1.0499E-05
TC2000008099.hg.1	UCKL1-AS1	1.8491625	0.88687201	6.75138442	3.9091309	0.002027741
TC1400010620.hg.1	SNAPC1	1.84618949	0.88455063	9.39386546	7.20710013	1.00265E-05
TC0600014102.hg.1	HCP5	1.84408844	0.88290785	7.7894498	8.2809397	2.41945E-06
TC0Y00006474.hg.1	CD99P1	1.79173154	0.84135449	8.26185847	5.56632785	0.00011694
TC1600007528.hg.1	ITGAM	1.79137733	0.84106926	5.71621081	5.32008032	0.000174577
TC0400008785.hg.1	NOCT	1.78987027	0.83985503	10.0042028	5.83425955	7.63514E-05
TC1900008496.hg.1	PPP1R15A	1.75637954	0.81260463	10.3916016	7.04712066	1.25409E-05
TC1200010341.hg.1	PKP2	1.75185809	0.80888592	9.29322269	5.8454491	7.50205E-05
TC0800012216.hg.1	CYHR1	1.73909696	0.79833837	12.2411329	6.92236269	1.49657E-05
TC1100011833.hg.1	SYTL2	1.7283526	0.78939757	9.78374147	6.80662615	1.76643E-05
TC0300009301.hg.1	PTX3	1.72781334	0.78894737	8.65423887	4.63064834	0.000560552
TC0100016901.hg.1	CACNA1S	1.70276159	0.76787646	5.53220612	4.81415575	0.000408349
TC1700012332.hg.1	SPATA22	1.70049442	0.76595428	4.41158956	5.35668517	0.000164393
TC1000007132.hg.1	BAMBI	1.66964303	0.73953969	9.1059875	4.5973759	0.000593974
TC0200014912.hg.1	DLX2	1.65920564	0.7304927	8.36939349	5.23102285	0.000202224
TC0500009357.hg.1	MIR103B1	1.65887038	0.73020116	4.05113064	5.33592343	0.00017009
TC0500012223.hg.1	NRG2	1.65677905	0.72838121	8.43223457	5.4722085	0.000136157

TC1600010174.hg.1	MYLK3	1.65448318	0.72638062	6.49741087	4.30890918	0.000987101
TC0X00006835.hg.1	MAGEB18	1.64038851	0.71403754	3.64297928	3.55906567	0.003857136
TC1700009670.hg.1	ALOXE3	1.63787572	0.71182589	6.51620873	4.27425292	0.001049928
TC0400008663.hg.1	HSPA4L	1.6358116	0.7100066	9.87371136	6.24788703	4.0322E-05
TC1100009514.hg.1	TMEM45B	1.62988157	0.70476714	5.09520063	4.23675609	0.001122593
TC1000008619.hg.1	NKX2-3	1.61191764	0.68877803	6.79428231	5.19962251	0.000213039
TC1700010604.hg.1	NR1D1	1.60863166	0.68583402	11.0960379	5.88411601	7.0607E-05
TC0100009959.hg.1	TUFT1	1.59316438	0.67189513	11.0108075	5.7484349	8.74272E-05
TC0400011616.hg.1	TIFA	1.58343008	0.66305316	7.59650026	3.77835702	0.002575215
TC0500008994.hg.1	SH3RF2	1.57281174	0.653346	7.83859088	4.30425115	0.000995313
TC1900006652.hg.1	TJP3	1.56126033	0.64271111	5.17503064	3.72396056	0.002845658
TC0400008106.hg.1	HERC5	1.54532989	0.62791485	9.19983697	4.60645493	0.000584653
TC1200010776.hg.1	KRT4	1.53699768	0.62011499	5.08848287	4.8446612	0.000387569
TC0300010770.hg.1	CSRNP1	1.53696014	0.62007975	7.66888812	4.94719311	0.000325468
TC0500012017.hg.1	IRF1	1.53619874	0.61936487	9.09677426	4.2947472	0.00101229
TC0200009376.hg.1	GPR39	1.51695799	0.60118113	8.07921316	3.61766518	0.003461202
TC1500009172.hg.1	MIR4310	1.51637537	0.60062693	4.21323151	4.11116516	0.001406275
TC1300009204.hg.1	KLHL1	1.51538787	0.5996871	4.6065569	3.84398017	0.00228371
TC1000008727.hg.1	NFKB2	1.51426015	0.59861308	9.98442603	5.27970949	0.000186582
TC0900009700.hg.1	IFNA14	1.51411895	0.59847855	6.40651451	4.21754982	0.001161812
TC1400010041.hg.1	IFI27L2	1.50696142	0.59164248	11.2270543	5.66963215	9.90965E-05
TC1100011182.hg.1	PYGM	1.47832821	0.56396661	5.01849805	3.55098811	0.003915226
TC1400007584.hg.1	TTC9	1.47383937	0.55957929	5.09815657	3.57139892	0.003770132
TC0100011282.hg.1	SOX13	1.47371868	0.55946116	10.3869961	5.18296064	0.000219023
TC0500012784.hg.1	LINC01187	1.45913823	0.54511657	4.62468148	3.84815666	0.002266346
TC1900011882.hg.1	ZNF442	1.42402332	0.50997277	7.97204819	3.64731727	0.003276917
TC1200007690.hg.1	TNS2	-1.4335619	-0.5196042	8.13487177	-4.1889005	0.001222971
TC1500009830.hg.1	LCTL	-1.4840043	-0.5694953	8.51683153	-4.9583356	0.000319377
TC0X00008188.hg.1	MIR448	-1.5414094	-0.6242501	4.55658969	-4.7575332	0.000450068
TC0100011472.hg.1	HHAT	-1.5505747	-0.632803	8.07158497	-4.4336857	0.000791397
TC0100006873.hg.1	RNU5E-1	-1.5778965	-0.6580026	9.62121242	-4.7855432	0.0004289
TC0800009764.hg.1	PSD3	-1.5792694	-0.6592573	9.04197934	-3.8323145	0.002332939
TC1000008643.hg.1	SCD	-1.5967151	-0.6751069	12.373124	-4.0278549	0.00163452
TC1200009807.hg.1	C3AR1	-1.6672495	-0.73747	6.39722751	-5.212152	0.000208653
TC0500007258.hg.1	GHR	-1.6714614	-0.74111	8.74000979	-4.7569127	0.000450549
TC1700010590.hg.1	GSDMB	-1.6880118	-0.755325	6.87474711	-4.5533925	0.000641372
TC1500006786.hg.1	SCG5	-1.7294924	-0.7903487	8.96346216	-5.8579975	7.35571E-05
TC0600012747.hg.1	CD24	-1.8341699	-0.8751273	11.1415955	-8.122591	2.95876E-06
TC2000007005.hg.1	CST7	-1.8555241	-0.8918268	8.18023389	-7.5294635	6.44972E-06
TC0800010774.hg.1	EYA1	-1.9620273	-0.9723451	11.4879738	-8.2624085	2.47675E-06
TC0800008140.hg.1	ATP6V0D2	-2.0331646	-1.023727	8.16892952	-6.2674057	3.91482E-05
TC1700012466.hg.1	LINC00673	-2.0390828	-1.0279204	8.72043514	-8.9786393	1.02946E-06
TC1000008955.hg.1	PLEKHS1	-2.0617416	-1.0438635	8.75069761	-7.359145	8.12983E-06
TC1000008099.hg.1	LRMDA	-2.0728323	-1.0516034	8.44997039	-6.9327484	1.47459E-05
TC1700010560.hg.1	PLXDC1	-2.1001812	-1.0705138	9.20252491	-8.895719	1.13649E-06
TC1100007400.hg.1	TSPAN18	-2.1173068	-1.0822303	9.5276138	-6.9848118	1.36949E-05
TC0100015714.hg.1	SV2A	-2.2532964	-1.1720371	11.0206927	-9.5782869	5.1369E-07
TC0X00010787.hg.1	APLN	-2.2829064	-1.1908717	10.212155	-7.7563397	4.76405E-06
TC0100015102.hg.1	COL11A1	-2.594436	-1.3754209	11.2614544	-9.1239352	8.67093E-07

TC0100012247.hg.1	KIF26B	-2.7419515	-1.455203	8.82455013	-8.5999678	1.62653E-06
TC1200011496.hg.1	DCN	-3.2816966	-1.7144418	8.23121539	-6.1743961	4.50874E-05

© 2020 by the authors. Submitted for possible open access publication under the terms and conditions of the Creative Commons Attribution (CC BY) license (http://creativecommons.org/licenses/by/4.0/).
